# Testing adaptive hypotheses on the evolution of larval life history in acorn and stalked barnacles

**DOI:** 10.1002/ece3.5645

**Published:** 2019-09-18

**Authors:** Christine Ewers‐Saucedo, Paula Pappalardo

**Affiliations:** ^1^ Department of Genetics University of Georgia Athens GA USA; ^2^ Odum School of Ecology University of Georgia Athens GA USA

**Keywords:** barnacles, brooding, comparative study, development, larvae, life history evolution, phylogenetics, Thoracica

## Abstract

Despite strong selective pressure to optimize larval life history in marine environments, there is a wide diversity with regard to developmental mode, size, and time larvae spend in the plankton. In the present study, we assessed if adaptive hypotheses explain the distribution of the larval life history of thoracican barnacles within a strict phylogenetic framework. We collected environmental and larval trait data for 170 species from the literature, and utilized a complete thoracican synthesis tree to account for phylogenetic nonindependence. In accordance with Thorson's rule, the fraction of species with planktonic‐feeding larvae declined with water depth and increased with water temperature, while the fraction of brooding species exhibited the reverse pattern. Species with planktonic‐nonfeeding larvae were overall rare, following no apparent trend. In agreement with the “size advantage” hypothesis proposed by Strathmann in 1977, egg and larval size were closely correlated. Settlement‐competent cypris larvae were larger in cold water, indicative of advantages for large juveniles when growth is slowed. Planktonic larval duration, on the other hand, was uncorrelated to environmental variables. We conclude that different selective pressures appear to shape the evolution of larval life history in barnacles.

## Graphical abstract

In the present study, we assessed if adaptive hypotheses explain the distribution of the larval life history of thoracican barnacles within a strict phylogenetic framework. In accordance with Thorson's rule, the fraction of species with planktonic‐feeding larvae declined with water depth and increased with water temperature, while the fraction of species with nonplanktonic larvae exhibited the reverse pattern. In agreement with the “size advantage” hypothesis proposed by Strathmann in 1977, egg and larval size were closely correlated to each other, and settlement‐competent cypris larvae were larger in cold water, indicative of advantages for large juveniles when low temperatures slow development and growth.

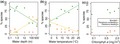

## 1 INTRODUCTION

Marine invertebrate larvae have long been used to study trait evolution (e.g., Darwin, 1851; Thorson, 1950). They have easily recognizable traits such as their size and feeding mode, which allow testing of a range of existing adaptive hypotheses. Moreover, the larval phase of marine benthic invertebrates is crucial for their fitness (Bownds, Wilson, & Marshall, 2010; Emlet & Sadro, 2006; Marshall & Keough, 2007), determining, for example, dispersal potential and fecundity. Despite this strong selective pressure to optimize larval life history, larvae of some taxa are curiously diverse even between closely related species, species that share the same adult life history or live in the same habitat. Examples of such phylogenetically labile taxa exist in molluscs (Collin, 2004; Duda & Palumbi, 1999; Liebermann, Allmon, & Eldredge, 1993; Pappalardo, Rodríguez‐Serrano, & Fernández, 2014), asterinid sea stars (Byrne, 2006; Hart, Byrne, & Smith, 1997), and echinoid sea urchins (Wray, 1996), with closely related species displaying divergent larval traits. This lability has been exploited extensively in comparative studies, which revealed the adaptive potential of larval life history. In contrast, the adaptive potential of less labile taxa has rarely been considered. Taxa with such a phylogenetically conserved larval life history seem to be, for example, thoracican barnacles (Barnard, 1924; Barnes & Barnes, 1965), pagurid hermit crabs (Nyblade, 1974), and temnopleurid echinoids (Jeffery & Emlet, 2003).

**Figure 1:**
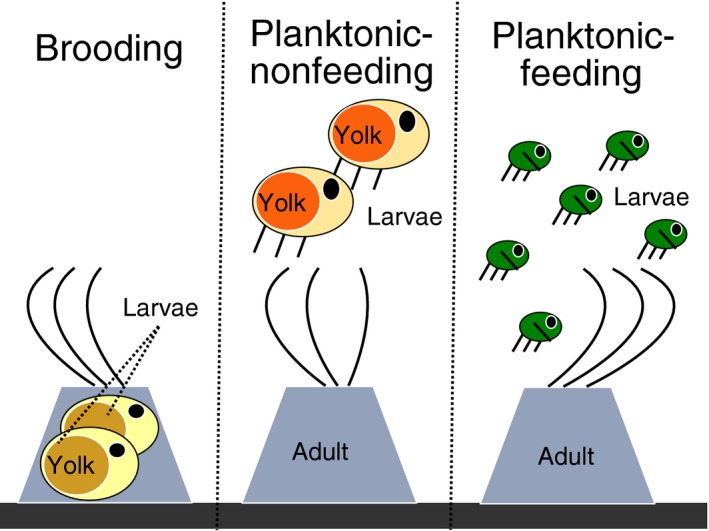
Characterization of larval developmental modes for thoracican barnacles. Larval modes are categorized as feeding or nonfeeding, and as planktonic or brooded. Feeding means that larvae are actively using food sources such as phytoplankton, and cannot complete their larval cycle without it. Nonfeeding larvae consume yolk provisioned by the mother, and complete their development without the need for external food sources. Planktonic larvae spend days to weeks in the water. Brooded larvae remain in the maternal individual up to the cypris stage, which settles shortly after release

Larval traits such as size, feeding mode, and the time larvae spend in the water to complete development (called planktonic larval duration or pelagic larval duration, PLD) are generally correlated and can be described by their developmental mode (Strathmann, 2007). In most marine invertebrates, larval development is either planktonic‐feeding (also called planktotrophic), planktonic‐nonfeeding (also called lecithotrophic), or benthic. In benthic development, the embryos are protected in special structures such as egg capsules or egg masses, or brooded by the female throughout most of the larval development (Figure 1; Strathmann, 2007; Thorson, 1950). Brooding species have large yolky eggs, the larvae do not feed and spend a negligible amount of time in the plankton. Planktonic‐nonfeeding larvae also hatch from large eggs and do not feed but can spend hours to weeks in the plankton, while planktonic‐feeding larvae spend weeks to months in the plankton acquiring food resources to complete development. Egg size differences result in the well‐known life history trade‐off between the size and number of eggs a female can produce, and consequently between fecundity and larval developmental mode (e.g., Marshall, Krug, Kupriyanova, Byrne, & Emlet, 2012; Thorson, 1950).

Several mathematical and conceptual models explore which environmental conditions select for different larval developmental modes and egg sizes (e.g., Christiansen & Fenchel, 1979; Strathmann, 1977; Thorson, 1950; Vance, 1973). The premise of one set of models is that by shortening the time larvae spend in the plankton, larvae could reduce planktonic predation risk (Hirst & Kiørboe, 2002; Morgan, 1995; Pechenik, 1999; Rumrill, 1990; Strathmann, 1985; White, Morgan, & Fisher, 2014). We refer to these models pioneered by Vance (1973) as “plankton‐duration” models. Their main assumptions are that egg size correlates to the length of the planktonic phase and that size at metamorphosis or settlement is independent of egg size (Christiansen & Fenchel, 1979; Levitan, 2000; Vance, 1973). In these plankton‐duration models, planktonic‐nonfeeding larvae develop faster than planktonic‐feeding larvae (Vance, 1973 [see Marshall & Keough, 2007 for discussion]; Christiansen & Fenchel, 1979; Havenhand, 1993; Levitan, 2000). Faster‐developing larvae are predicted to be selected for at low temperatures, where development is slowed down (O'Connor et al., 2007), and an extended planktonic phase increases absolute exposure to predators. Similarly, low food availability should select for nonfeeding larvae. In line with these expectations, experimental rearing studies on barnacle larvae showed that low food availability increased mortality of planktonic‐feeding larvae (Anil, Desai, & Khandeparker, 2001; Zabin, Zardus, Pitombo, Fread, & Hadfield, 2007). In summary, the plankton‐duration models predict that feeding larvae become less abundant as temperature and food availability decrease, while nonfeeding larvae (brooded and planktonic), which develop relatively faster than planktonic‐feeding larvae, are more abundant at lower temperatures and lower food availability. This explanation for the latitudinal distribution of developmental modes was proposed by Thorson (1950), and is known as Thorson's rule.

Thorson's rule has been tested extensively using community or comparative datasets, and explains the geographic distribution of larval developmental mode in gastropods, bivalves, chitons, octopuses, anomurans, brachyurans, peracarids, holothuroids, and ophiuroids (e.g., Collin, 2003; Fernández, Astorga, Navarrete, Valdovinos, & Marquet, 2009; Pappalardo & Fernández, 2014; Ibáñez et al., 2018; see table 1 in Ibáñez et al., 2018 for a detailed compilation of studies analyzing Thorson's rule). Interestingly, several other studies, sometimes on the same taxa, did not find support for Thorson's rule (e.g., Pearse, 1994 for holothuroids and ophiuroids, Voight, 1998 for octopuses and Stanwell‐Smith, Peck, Clarke, Murray, & Todd, 1999 for Antarctic invertebrates). Ibáñez et al. (2018) suggest that these contrasting findings are the result of different analytical approaches, and a general lack of phylogenetic statistics (but see Pappalardo et al., 2014).

Selection may also, or alternatively, operate on egg size. Large larvae might be better equipped to feed on large phytoplankton (Barnes & Barnes, 1965), and large juveniles have higher survival rates (Emlet & Sadro, 2006; Gosselin & Qian, 1996; Hunt & Scheibling, 1997; Miller & Carefoot, 1989; Moran & Emlet, 2001; Spight, 1976; Strathmann, 1977). We refer to these strategies first conceptualized by Strathmann (1977) as “size‐advantage” models, which assume that egg size is positively correlated with larval size and size at metamorphosis or settlement, and that planktonic larval duration is independent of egg size (Strathmann, 1977). The premise of these models is that eggs should be large when it is advantageous to have large larvae or large juveniles (Strathmann, 1977). One prediction is that eggs and the resulting feeding larvae are larger in cold water in order to feed on the large phytoplankton typical for cold climate (Barnes & Barnes, 1965). Few other concrete predictions for environmental selective agents exist (see e.g., Marshall & Keough, 2007), and we therefore correlate size of early larvae and settlement‐competent larvae, a proxy for juvenile size, to different environmental variables.

Patterns of larval development in deep sea species might also be related to dispersal or colonization potential (Rex & Warén, 1982). A high potential for dispersal may be beneficial to colonize patchily distributed or ephemeral habitats, which are frequent in the deep sea (Buhl‐Mortensen & Høeg, 2006). In particular, long planktonic durations might be advantageous at seamounts, where mesoscale flow can form eddies that retain larvae near the seamount even if they spend a long time in the plankton. If eddies are detached, the larvae can also be transported long distances. In consequence, a long planktonic phase facilitates both local recruitment and colonization of new seamounts (Mullineaux, 1994). In hydrothermal vents, which are temporally unstable, larvae can be transported long distances in the buoyant plumes, which may allow them to colonize new vents (Mullineaux, 1994). These different hypotheses lead to the overall prediction that a high proportion of species have planktonic larvae in the deep sea. Evidence from bivalves and gastropods support this hypothesis, but suggest that the pattern may also be determined by taxonomic composition (Scheltema, 1994).

Thoracican barnacles are a well‐studied and widely‐distributed crustacean taxon with diverse life histories (Anderson, 1994; Darwin, 1851, 1854; Newman & Ross, 1971), which makes them a good model to test adaptive hypotheses. However, barnacle larvae have been considered phylogenetically constrained, and lacking adaptive potential (Barnard, 1924; Barnes & Barnes, 1965). Our first goal is therefore to formally test the strength of the phylogenetic signal for larval traits, and assess if the evolution of larval life histories is phylogenetically constrained. Our second goal is to assess whether the assumptions (specific correlations among larval traits) and predictions (correlations between larval traits and environmental variables) of the different adaptive hypotheses are met. In order to achieve these goals, we searched the literature for larval life history data from thoracican species. From publicly available information on species occurrences and environmental remote‐sensing data, we compiled information on depth, ocean temperature and chlorophyll *a* concentration, the latter being a proxy for food availability of shallow‐water invertebrates (Lorenzen, 1970; Marshall et al., 2012). We accounted for phylogenetic nonindependence using the newly synthesized barnacle tree of life (Ewers‐Saucedo et al., 2019).

## 2 MATERIAL AND METHODS

### 2.1 Study organisms

The thoracican barnacles are an abundant taxon of marine crustaceans (Newman & Abbott, 1980). Most species are sessile hermaphrodites, which copulate with long penises (Anderson, 1994). Eggs develop in the female body cavity, and free‐swimming naupliar larvae are released into the water; these planktonic larvae spent several days to weeks in the water column, and can be nonfeeding or feeding (Figure 1). The typical larval development in barnacles proceeds through six naupliar stages, followed by the cypris stage (a nonfeeding final stage competent to settle). After settlement, the cyprid metamorphoses into the juvenile form. Cypris larvae can both swim and crawl (Høeg & Møller, 2006). Their dispersal distance is much lower than that of nauplius larvae (Barnard, 1924; Høeg & Møller, 2006; Southward, 1998). In some species with nonfeeding larvae, the embryos are brooded by the maternal individual and hatch as cypris larvae into the water column. We call this third larval developmental mode “brooded” and speak of brooding species to allow comparisons to other invertebrate taxa (Figure 1).

### 2.2 Life history data

We compiled information on early life history traits by searching for relevant publications (http://www.scholar.google.com, accessed November 2016) with the following terms: “Cirripedia”, “Thoracica”, “barnacle”, “egg size”, “cypris size”, “larval duration”, and “larval development.” We classified the mode of larval development as brooding, planktonic‐nonfeeding, or planktonic‐feeding as described in the previous section (Figure 1). From laboratory‐rearing studies, we collected information on the planktonic larval duration (PLD), the rearing temperature, length of the egg, first naupliar, and cypris stage, as well as latitude, longitude, and depth of the collection location. Egg length and length of the first naupliar stage have a strong positive correlation in thoracican barnacles. When only the size of the first naupliar stage was reported, we used a linear regression model to predict egg size based on naupliar size (see Appendix S1 for details).

Temperature has a large effect on the PLD of marine larvae (O'Connor et al., 2007), and thus larvae reared under different temperatures will differ in PLD. To account for these differences, we normalized PLD to 20°C based on *Q*_10_ values as in Levitan (2000; see Appendix S2 for details). In several species, different studies measured egg size or PLD. We therefore calculated mean and standard error for all egg size and PLD estimates by species, and used the standard error to account for trait variance. Where only one measurement per species was available, we used the median of all standard errors to approximate trait variance. Several authors have pointed out intraspecific latitudinal gradients in egg size (Clarke, 1992; Marshall et al., 2012). In barnacles, however, such local variation appears largely absent (Barnes & Barnes, 1965). We confirmed this observation, showing that egg size increased with latitude only in *Semibalanus balanoides* (Linnaeus, 1767; see Appendix S3 for details). Normalized PLD did not show intraspecific latitudinal trends either.

### 2.3 Occurrences and environmental data

We used occurrence data (geographic coordinates) with two aims: (a) to get additional information on the depth distribution for the species with life history information, and (b) to extract information on environmental conditions at the locations of occurrence of the species. For all the species with life history information, we extracted occurrence data from three sources, (a) the Ocean Biogeographic Information System (OBIS: http://www.iobis.org), (b) the Global Biodiversity Information Facility (GBIF: http://www.gbif.org), and (c) from collection points reported in the primary literature. We searched for occurrence data using the taxonomic names published in the Open Tree of Life (Hinchliff et al., 2015), as well as their synonyms available in the World Register of Marine Species (WoRMS Editorial Board, 2015, http://www.marinespecies.org). We detail how we combined occurrences and extracted environmental data in Appendix S4.

We searched for information on ocean temperature, chlorophyll *a* concentration, and water depth for each occurrence in our database. In situ seasonal temperature was downloaded as a NetCDF file from the World Ocean Atlas 2013 (http://www.nodc.noaa.gov/cgi-bin/OC5/woa13/woa13.pl), with a resolution of 1/4°. SeaWiFS (spatial resolution: 9 km) estimates of chlorophyll *a* concentration were downloaded as NetCDF file from the Giovanni online data system (https://giovanni.sci.gsfc.nasa.gov/giovanni/). Chlorophyll *a* concentration was used as a proxy for phytoplankton availability (Lorenzen, 1970) only in shallow water species (<30 m).

To interpolate the gridded oceanographic data to our occurrences, we used the function “nn2” in the “RANN” package (Arya, Mount, Kemp, & Jefferis, 2019). From all occurrences for each species, we calculated the median value of each environmental variable and the interquartile range to represent variation. When we could not compute the interquartile range due to insufficient data, we used the overall median of interquartile ranges from all species with this information.

### 2.4 Phylogenetic analyses

All comparative data analyses were carried out in the R environment version 3.3.1 “Bug in Your Hair” (R Development Core Team, 2016), using the packages “ape” v. 3.5 (Paradis, Claude, & Strimmer, 2004), “caper” v. 0.5.2 (Orme et al., 2013), “geiger” v. 2.0.6 (Harmon, Weir, Brock, Glor, & Challenger, 2008), “nlme” v. 3.1‐128 (Pinheiro, Bates, DebRoy, Sarkar, & R Core Team, 2019), “phytools” v. 0.5‐38 (Revell, 2012), “phylolm” v. 2.5 (Ho & Ané, 2014), and “sensiPhy” v. 0.5‐0 (Paterno, Werner, & Penone, 2018). Recent efforts have combined all phylogenetic reconstructions available for Thecostraca, which includes the thoracican barnacles, with a taxonomic backbone to generate a complete phylogenetic hypothesis for Thecostraca (Ewers‐Saucedo et al., 2019). This synthesis tree does not provide meaningful branch lengths. We used congruification (Eastman, Harmon, & Tank, 2013) to time‐calibrate the tree based on the age of several nodes common to the synthetic phylogeny and chronograms available for Cirripedia and Balanomorpha (Pérez‐Losada et al., 2008, 2014) by using the function “congruify.phylo” in the R package “geiger.” After identifying 44 common calibration nodes, we transformed branches of the synthetic tree with the semiparametric method of Sanderson (2002) based on penalized likelihood, implemented in the function “chronos” in the R library “ape.” This method controls mutation rate variation among branches using a smoothing parameter lambda. Given that branch lengths in the synthetic tree do not have biological meaning, for example, do not represent differentiation between species, we set lambda to zero. This allowed uncorrelated branch length variation.

#### 2.4.1 Phylogenetic signal

We evaluated the phylogenetic signal of larval developmental mode by calculating the phylogenetic *D* statistic for binary traits (Fritz & Purvis, 2010), and the phylogenetic signal of egg size and PLD by calculating Blomberg's *K* (Blomberg, Garland Jr, & Ives, 2003). To calculate the phylogenetic *D* statistic, we coded larval modes as binary by considering whether nauplius larvae were planktonic or brooded, and whether they were actively feeding or not. We tested for significant departures from both random association and the clumping expected under a Brownian evolution threshold model using randomization tests with 1,000 permutations (function “phylo.d,” R package “caper”). *D* values of one indicate the absence of phylogenetic signal, values of zero indicate that the binary trait evolves under Brownian Motion, and values smaller than 0 signify highly conserved traits. We tested whether the continuous traits PLD and egg size evolved under the process of Brownian Motion (BM) by calculating Blomberg's *K* (Blomberg & Garland Jr, 2002; Blomberg et al., 2003) with the function “phylosig” in the R package “phytools.” Traits evolve under BM when Blomberg's *K* equals one *K* larger than one suggests that traits are more similar than expected under neutral evolution, while *K* smaller than one suggests that closely related species are less similar than expected under BM. The latter pattern might be an indication of adaptive evolution (Blomberg et al., 2003). The significance of *K* was evaluated based on the variance of phylogenetically independent contrasts relative to tip shuffling randomization. This analysis required a completely bifurcating tree, and we randomly resolved polytomies.

#### 2.4.2 Ancestral state reconstruction

We reconstructed ancestral larval modes for all nodes based on the best‐fitting model of trait evolution (see Appendix S7: “Models of trait evolution”) using the maximum likelihood approach implemented in “ace” in the R package “ape.” The analysis required a completely bifurcating tree and nonzero branch lengths, and we randomly bifurcated unresolved nodes (function “multi2di”, R package “ape”), and replaced zero branch lengths with a small value (0.1).

#### 2.4.3 Correlations among larval traits

We fit phylogenetic generalized least squares (PGLS) models between egg size and normalized PLD, size of the first nauplius larvae, and cypris size, while taking larval mode into account. We also tested for size differences of eggs and cyprids with different larval modes with PGLS, as well as between PLD and larval modes. In all PGLS analyses, we modeled covariance based on the best‐fitting model of trait evolution (see Appendix S7: “Models of trait evolution”) and took trait variation into account (function “gls,” R package “nlme”). We excluded brooding species from the analyses of PLD, as the PLD for brooded larvae in barnacles is presumably close to zero (Barnard, 1924; Southward, 1998). We also tested for a nonlinear relationship between egg size and PLD, as proposed by Levitan (2000; see Appendix S5). Levitan suggested that PLD is proportional to egg volume rather than linearly related to egg diameter.

#### 2.4.4 Correlations between larval traits and environmental variables

We only assessed the effects of single environmental variables on larval traits due to a strong collinearity between water depth and temperature, as well as temperature and chlorophyll *a* concentration (Pearson's correlation coefficients between 0.69 and 0.74, see Appendix S6 for details). Remote‐sensing chlorophyll *a* data represent an integrated estimate across shallow depth layers, and was only analyzed for shallow water species. We tested the effects of depth, water temperature, and chlorophyll *a* concentration on larval mode with Phylogenetic Generalized Linear Mixed Models for Binary Data, implemented in the function “binaryPGLMM” in the R package “ape.” For logistic regressions, we coded larval mode as binary based on (a) the presence or absence of a planktonic phase, and (b) the feeding mode (Figure 1). Lastly, we tested whether egg size, cypris size, or PLD varied with any of the environmental variables using PGLS models, taking larval development and variance for environmental variables into account (function “gls” in the R package “nlme”).

#### 2.4.5 Larval mode composition along environmental gradients

We assessed differences in the composition of species with each larval developmental mode along environmental gradients for depth, temperature, and chlorophyll *a*. To do so, we split each environmental variable into five to eight categories that contained the same number of species, irrespective of their larval mode. For each larval mode, we then performed linear regression between the midpoint values of the environmental variable bins and the fraction of species with a particular larval mode.

## 3 RESULTS

### 3.1 Life history data

**Figure 2:**
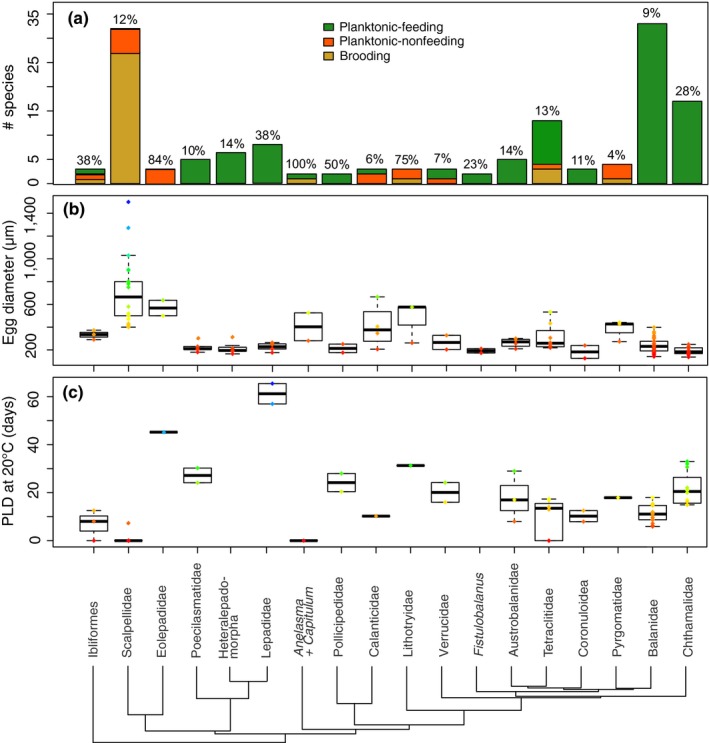
Clade‐specific summary of larval traits and data availability. (a) Distribution of larval developmental modes in each clade. Noted above each bar is the percentage of species with available larval trait information. (b) Distribution of egg sizes in each clade. (c) Distribution of planktonic larval duration (PLD) normalized to 20°C in each clade. The clades represented are often families when those were mostly monophyletic. In some cases, recent phylogenetic results excluded certain genera from their taxonomic families. Those genera are represented as separate branches. In particular, these are *Anelasma*, *Capitulum*, and *Fistulobalanus*. Similarly, some families do not appear to be monophyletic, and dissipate into larger families. In particular, the family Archaeobalanidae merges with the Balanidae, the Catophragmaidae with the Chthamalidae and the Pachylasmatidae with the Tetraclitidae. Lastly, some families with few species were combined into larger monophyletic taxa. This was done for the families Iblidae and Iblioidae, which were summarized in the Ibliformes, the families Chelonibiidae, Coronulidae and Platylepadidae, which were combined in the Coronuloidea, and the families Heteralepadidae, Koleolepadidae, and Microlepadidae were merged into the Heteralepadomorpha. We were unable to obtain larval trait information for the families Chionelasmatidae (four species total), Malacolepadidae (two species), Oxynaspididae (28 species), and Rhizolepadidae (two species). (b) and (c) are box plots where the thick line indicates the median, the box indicates the 25 and 75 percentiles and the whiskers extend to the most extreme data point which is 1.5 times the interquartile range from the box

We collected life history information for 170 species that represent most families in the Superorder Thoracica (Figure 2). The percentage of species included from each of the families ranged from 4% to 100% (Figure 2a). We were unable to obtain larval trait information for the small families Chionelasmatidae, Malacolepadidae, and Rhizolepadidae, which contain less than five species each, and the Oxynaspididae, which contains 28 nominal species. Most data were available for larval developmental mode (*n* = 153 species; Figure 2a). Planktonic‐feeding larvae were reported for 103 species (67% of all species), planktonic‐nonfeeding larvae for 19 species (12%), and brooded larvae for 33 species (22%). Information on the size of egg or first nauplius (*n* = 136 species), cypris size (*n* = 93), and PLD (*n* = 90 species) was available for fewer species. Egg size ranged from 107 μm to 1,500 μm, with an average standard error of 21 μm per species (Figure 2b), cypris size from 375 μm to 2,550 μm (average standard error per species = 19 μm), uncorrected PLD ranged from 4 to 110 days, and *Q*_10_‐corrected PLD for 20°C from 4.5 to 58.12 days, with an average standard error of 0.72 days per species (Figure 2c). The variation in egg size and temperature‐corrected PLD within clades is shown in Figure 2, while species‐specific information is available online should the manuscript be accepted. For the review process, the information is available as supplementary file.

### 3.2 Environmental data

We obtained environmental data for all species but *Dichelaspis darwini* (Philippi, 1861). From 21,844 geo‐referenced occurrences, we compiled depth information for 169 species, temperature for 150 species, and chlorophyll *a* for 85 shallow‐water species (<30 m). Of the shallow‐water species, 77 had planktonic‐feeding larvae, seven had planktonic‐nonfeeding larvae and four had brooded larvae. The number of depth observations per species ranged from one for *Paralepas scyllarusi* Utinomi, 1967, which we obtained from the primary literature, to 4,262 for *Balanus crenatus* Bruguière, 1789. The median number of occurrences per species was 10, the mean was 98. The distribution of depth, temperature, and chlorophyll a for each larval development is displayed in Figure 5.

### 3.3 Phylogenetic analyses

#### 3.3.1 Phylogenetic signal

All the larval traits analyzed showed a phylogenetic signal. The *D* statistic for feeding mode was −0.610, suggesting that feeding mode was highly conserved and more similar than expected under BM. Similarly, the D statistic for the presence or absence of a planktonic phase was −0.627. Egg size and normalized PLD also had significant phylogenetic signals: Blomberg's *K* was 0.497 for egg size, which was significantly different from zero (*p*‐value = .001), the null expectation in the absence of phylogenetic signal. Similarly, Blomberg's *K* for PLD was also significantly different from zero with 0.407 (*p*‐value = .003).

#### 3.3.2 Ancestral state reconstruction

**Figure 3:**
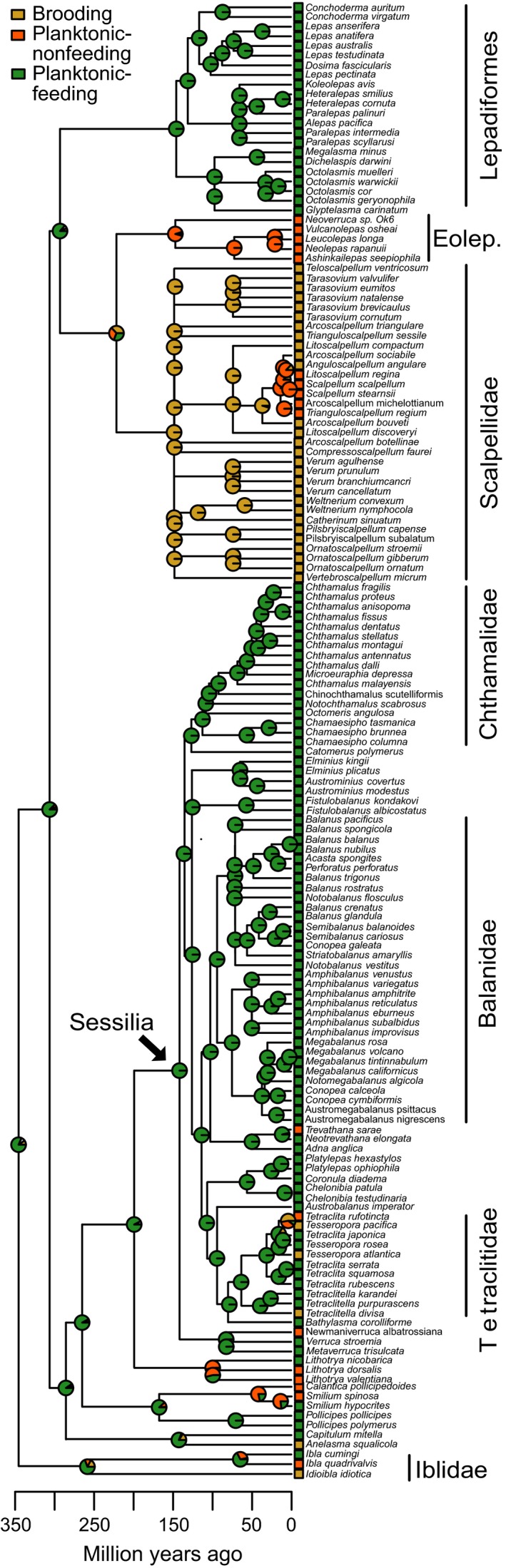
Maximum likelihood ancestral state reconstruction of modes of larval development of thoracican barnacles mapped onto the phylogeny, based on equal transition rates among all larval modes. Only families and orders mentioned throughout the text are indicated in the tree. The order Lepadiformes contains the families Lepadidae, Poecilasmatidae, and Heteralepadidae, which are shown in Figure 2. The genera *Conopea* and *Acasta* do not formally belong within the Balanidae, but phylogenetic reconstructions indicate their placement within this family. Abbreviations: Eolep., Eolepadidae

Reconstructing the ancestral state at the root of the tree based on this lambda‐transformed tree indicated that the ancestral thoracican barnacle had planktonic‐feeding larvae with a probability of 0.957 (Figure 3). Transitions between larval modes were uncommon. One major transition from planktonic‐feeding to brooded larvae occurred at the base of the Scalpellidae (Figure 3), with transitions to planktonic‐nonfeeding larvae in a subsequent clade of scalpellids. No re‐acquisition of the feeding habit was reconstructed with certainty. Several families had no variation in larval mode (Figure 3). Within the Order Sessilia (indicated with an arrow in Figure 3), changes from planktonic‐feeding to planktonic‐nonfeeding or brooding appeared more recently.

#### 3.3.3 Correlations among larval traits

**Figure 4:**
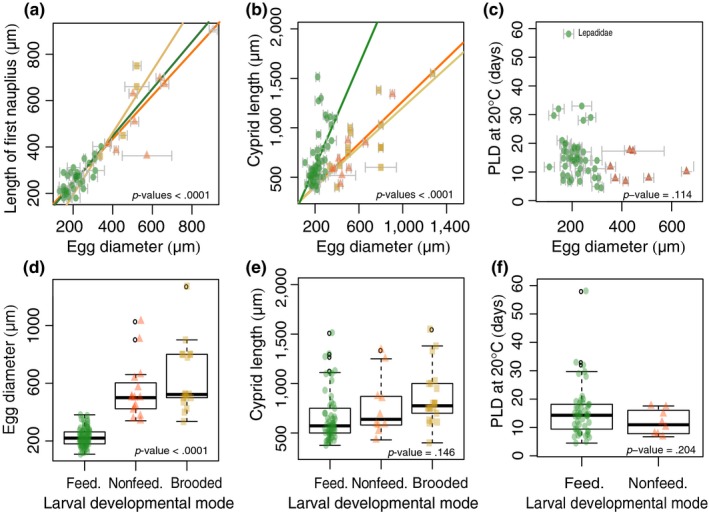
Relationships among larval traits. (a) Relationship between egg size and size of the first larval stage. (b) Relationship between egg size and cypris size; cyprids are the last larval stage, competent to settle and metamorphose into juveniles. (c) Relationship between egg size and planktonic larval duration (PLD). (d) Relationship between larval developmental mode and egg size. (e) Relationship between larval mode and cypris size. (f) Relationship between larval mode and planktonic larval duration (PLD). PLD was normalized to 20°C based on metabolic rate increase (Q_10_). We plotted the actual data points as colored shapes in both scatter plots and box plots, which refer to different larval modes (orange triangles: planktonic‐nonfeeding, green circles: planktonic‐feeding, yellow squares: brooding). In the box plot, the horizontal distribution of the actual data points is random, facilitating the plotting of many data points. The thick lines in the box plot indicate the median, boxes delimit 25 and 75 percentiles and whiskers extend to the most extreme data point which is 1.5 times the interquartile range. Abbreviations: brooded, brooding larval mode; feed., planktonic‐feeding larval mode; nonfeed., planktonic‐nonfeeding larval mode

Egg size and size of the first nauplius larva were significantly correlated in each larval mode (*p*‐value < .0001 for all larval modes; Figure 4a). Egg size and cypris size were well correlated within each larval mode (*p*‐values < .0001 for all larval modes). Specifically, the slope between egg size and cypris size did not differ for planktonic‐nonfeeding and brooded larvae, but was significantly steeper for planktonic‐feeding larvae (*p*‐value of the interaction term = .0013; Figure 4b). PLD was not shorter in larger eggs (*p*‐value = .114; Figure 4c), nor was egg volume correlated to Levitan's “T” (*p*‐value = .264), suggesting that egg size scales neither linearly nor proportionally with PLD. Egg size differed significantly between all larval modes (*p*‐value < .0001; Figure 4d). Cyprids did not differ in size between larval modes (*p*‐value = .146; Figure 4e). PLD did not differ between planktonic‐feeding and planktonic‐nonfeeding larvae (*p*‐value = .204; Figure 4f).

#### 3.3.4 Correlations between larval traits and environmental variables

**Figure 5:**
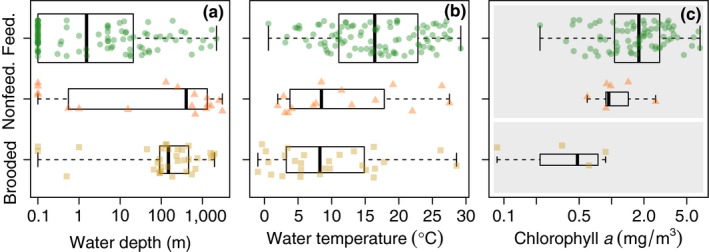
Relationships between larval developmental mode and the environmental variables (a) water depth, (b) water temperature, and (c) chlorophyll *a* concentration. Chlorophyll *a* concentration and water depth were plotted on a log‐scale. We show chlorophyll *a* data for shallow water species (<30 m water depth) only. Gray boxes indicate significant correlations between larval mode and the environmental variable. All figures are box plots where the thick line indicates the median; the box indicates the 25 and 75 percentiles; and the whiskers extend to the most extreme data points. We also plotted the actual data points as colored shapes (orange triangles: planktonic‐nonfeeding, green circles: planktonic‐feeding, yellow squares: brooding) over the box plots with a random vertical distribution

**Figure 6:**
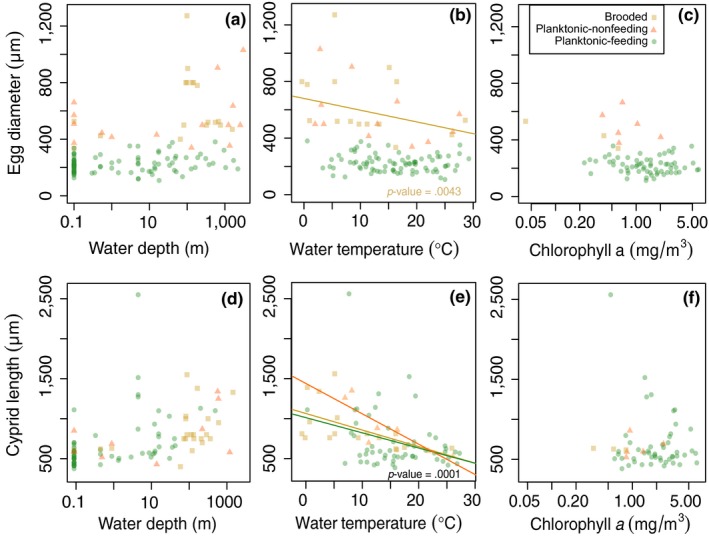
Relationships between egg size (a–c), size of the last larval stage (d–f), and environmental variables. The linear models used to test for significant relationships incorporated standard errors of the environmental variables, but these are omitted for legibility. A solid line indicates a significant relationship. Colors and shapes of the data points denote the larval developmental mode: orange triangles: planktonic‐nonfeeding, green circles: planktonic‐feeding, yellow squares: brooding

Depth did not correlate significantly with the presence of a planktonic phase (*p*‐value = .550) nor feeding mode (*p*‐value = .441; Figure 5a). Temperature was also uncorrelated with the distribution of planktonic versus brooded larvae (*p*‐value = .675) nor feeding versus nonfeeding larvae (*p*‐value = .956; Figure 5b). Species with planktonic larvae occurred in higher chlorophyll *a* concentrations (*p*‐value = .041; Figure 5c). Eggs of species with planktonic‐nonfeeding larvae were smaller at higher temperatures (*p*‐value = .0043; Figure 6b), but we did not observe this relationship for species with planktonic‐feeding larvae (*p*‐value = .124) or brooded larvae (*p*‐value = .651). Cyprids of all larval modes were significantly larger in colder water (*p*‐value = .0001; Figure 6e). Neither depth nor chlorophyll *a* concentration had a significant effect on egg size (*p*‐values = .339 and .394, respectively; Figure 6a,c) or cypris size (*p*‐values = .672 and .892, respectively; Figure 6d,f). PLD did not vary significantly with water depth (*p*‐value = .080), temperature (*p*‐value = .329), or chlorophyll *a* concentration (*p*‐value = .256).

#### 3.3.5 Larval mode composition along environmental gradients

**Figure 7:**
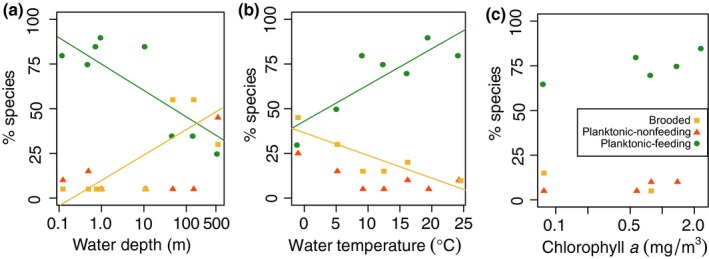
Relative distribution of larval developmental modes along environmental gradients for (a) water depth, (b) water temperature, and (c) chlorophyll *a* concentration. Water depth and temperature were split up into seven categories with 19–20 species per category, and chlorophyll *a* concentration into five categories with 17 species per category. The *x*‐axis values are the midpoints of each category. We fit linear regression models to the environmental distribution of each larval developmental mode. Fitted lines denote significant relationships. Colors and shapes of the data points denote the larval developmental mode: orange triangles: planktonic‐nonfeeding, green circles: planktonic‐feeding, yellow squares: brooding

The fraction of species with planktonic‐feeding larvae declined with water depth (*p*‐value = .0056), and increased with water temperature (*p*‐value = .0174; Figure 7a,b). This pattern was reversed for brooding species, which became more abundant in deeper water (*p*‐value = .025), and colder water (*p*‐value = .027; Figure 7a,b). The fraction of species with planktonic‐nonfeeding larvae did not correlate with any of the environmental variables (Figure 7c), and planktonic‐feeding larvae were most common at all chlorophyll *a* categories (*p*‐value = .112).

## 4 DISCUSSION

In concordance with Thorson's rule and the predictions of adaptive plankton‐duration models, the distribution of barnacle larval modes correlates with temperature: brooded larvae are common in cold water, while planktonic‐feeding larvae dominate warm water. At first glance, the ancestral trait reconstruction suggests deep developmental constraints of larval developmental mode, as evident by a striking phylogenetic bimodality: The majority of Sessilia have planktonic‐feeding larvae, while most Scalpellidae are brooding. However, transitions to other larval developmental modes have occurred in both taxa (Figure 3). The two scalpellids *Scalpellum scalpellum* (Linnaeus, 1767) and *Arcoscalpellum michelottianum* (Seguenza, 1876), for example, have planktonic larvae (Buhl‐Mortensen & Høeg, 2006; Svane, 1986; Spremberg, Høeg, Buhl‐Mortensen, & Yusa, 2012). These transitions, albeit infrequent, highlight that transitions between modes of larval development are possible in both directions. Just as noted by Thorson (1950) for other invertebrates, planktonic‐nonfeeding larvae are rare in barnacles (12% of all included species), and their presence is not correlated to the environmental gradients we investigated. Our data indicate why planktonic‐nonfeeding larvae may be rare: Species with planktonic‐nonfeeding larvae have large eggs (as expected), thus low fecundity in comparison with species with planktonic‐feeding larvae, but they do not develop faster than planktonic‐feeding larvae. In other taxa, for example, echinoids, planktonic‐nonfeeding larvae do develop faster than planktonic‐feeding larvae (Levitan, 2000). Thus, while planktonic‐nonfeeding larvae of echinoids likely have an advantage in cold water, where they can complete development faster than planktonic‐feeding larvae, planktonic‐nonfeeding larvae of barnacles do not seem to have this advantage.

The correlation between larval mode and temperature is not as clear cut as originally proposed by Thorson (1950), but agrees with more recent evaluations of Thorson's rule (e.g., Mileikovsky, 1971; Pearse, 1994). Neither water depth nor temperature was significantly correlated with larval mode in the logistic regression analyses, but showed significant shifts in the fraction of species along environmental gradients. Any larval mode is present at least at low frequencies under all environmental conditions, which can explain the nonsignificant results of the logistic regressions. Biologically, gradual shifts in the number of species of each larval developmental mode could be due to taxon‐specific tolerances to starvation and low temperatures. Where the planktonic‐feeding larvae of some species already starve or develop too slowly, others still thrive. In barnacles, these tolerances appear to have phylogenetic underpinnings. For example, genera of the deepest species with planktonic‐feeding larvae, *Metaverruca*, *Bathylasma*, and *Glyptelasma*, are all limited to the Arctic or deep sea (Newman & Ross, 1971). Their larvae might have evolved unique mechanisms to feed in environments with restricted phytoplankton availability, and cope with extended planktonic larval durations. Shallow‐water species with brooded or planktonic‐nonfeeding larvae also occur, especially in the Tetraclitidae and Iblidae. These larval modes evolved relatively recently within these taxa (see Figure 3), and occur in the same shallow and warm water as other members of these families. Other selective forces might be responsible for at least some of the transitions to planktonic‐nonfeeding larvae, such as habitat availability, adult size, or sexual system (Barnes, 1989; Strathmann & Strathmann, 1982). We conclude that different selective forces might be acting at different levels: at a broad phylogenetic scale, temperature, and food availability determine larval developmental mode, while at the family level or below, other forces might be important.

We did not find a significant relationship between the relative abundance of planktonic‐feeding larvae and chlorophyll *a* concentration. The notion that planktonic‐feeding larvae die of starvation at low phytoplankton concentrations is contentious (e.g., Vance, 1973), and has already been questioned by Thorson (1950). The logistic regression analysis indicated that the presence of a planktonic phase for both feeding and nonfeeding larvae was negatively correlated to chlorophyll *a* concentration. While the effect of chlorophyll *a* on the presence of planktonic larvae might be an artifact of the low number of barnacle species with planktonic‐nonfeeding larvae, Marshall et al. (2012) obtained the same result in a larger dataset including molluscs, echinoderms, and annelids. Chlorophyll *a*, or a correlated environmental factor, seems to favor planktonic larvae on a broad phylogenetic scale, rather than for feeding larvae in particular. It might be that chlorophyll *a* concentration is correlated to a factor that makes dispersal more advantageous, as species with planktonic larvae disperse further (e.g., Pannacciulli, Manetti, & Maltagliati, 2009; Scheltema, 1986). Future models need to evaluate under which scenarios we might expect this phylogenetically widespread pattern.

Thorson (1950) suggested that deep sea species should abandon the planktonic larval phase, a hypothesis that has recently been discounted (Clarke, 1992; Pearse, 1994; Stanwell‐Smith et al., 1999). Our data, on the one hand, show that the fraction of brooding species increases with water depth, as predicted by Thorson. On the other hand, barnacle species with planktonic larvae are more abundant in the deep sea than brooding species: of the 20 species with the deepest occurrence records, occurring between 558 m and 3,050 m, 14 species have planktonic larvae, while only six species are brooding. This result is mainly due to the abundance of species with planktonic‐nonfeeding larvae in the deep sea. The fraction of species with planktonic‐feeding larvae, however, decreases steadily with water depth. These results are comparable to patterns observed in echinoderms (Pearse, 1994) and molluscs (Scheltema, 1994). Half of the barnacle species with planktonic‐nonfeeding larvae belong to the family Eolepadidae, which live on deep‐water hydrothermal vents (Southward & Jones, 2003). Planktonic‐nonfeeding larvae might be advantageous in these temporally unstable habitats, where dispersal to other vents becomes crucial for the maintenance of the population (Yorisue et al., 2013). A long planktonic phase also facilitates both local recruitment and colonization in seamounts habitats (Mullineaux, 1994).

Strathmann (1977) pointed out that, regardless of egg size, all barnacles have six naupliar stages and that the larval phase is never abbreviated; therefore, he did not expect larger eggs typical for planktonic‐nonfeeding species to have a shorter planktonic phase. Our results confirm Strathmann's conclusion. In addition, egg size was closely correlated to the size of early nauplius larvae and settlement‐competent cypris larvae within each larval mode, matching the assumptions underlying Strathmann's size advantage hypothesis. Our data do not support the prediction that planktonic‐feeding nauplius larvae are larger in cold water in order to feed on larger phytoplankton (Barnes & Barnes, 1965), since only nonfeeding nauplius larvae (both planktonic and brooded) were larger at low temperatures. Interestingly, temperature correlated negatively with cypris size for all larval modes, which highlights the potential advantage for large juveniles in cold environments. Larger juveniles might have an advantage in cold climates because growth is slow at low temperatures (O'Connor et al., 2007), and larger juveniles can reach a size‐refuge from predation and dislodgement faster than their smaller counterparts (Ghiselin, 1987; Pearse, McClintock, & Bosch, 1991). This also provides an explanation for the negative correlation between egg size of nonfeeding larvae and temperature: nonfeeding larvae grow little throughout their development (Barnes & Achituv, 1981). In order to have large juvenile offspring, mothers need to produce large eggs. Feeding nauplius larvae, on the other hand, might achieve temperature‐dependent size differences at settlement by growing differentially throughout their larval development.

Our data originate from various studies, leaving room for inconsistencies and biases. More generally, the PLD estimates used in this study were obtained in laboratory‐rearing studies, and laboratory conditions may lead to unnatural results. On the one hand, larvae might be reared under suboptimal conditions, for example, with regard to their food requirements (Moyse, 1963; Strathmann, 1985). In this case, PLDs for planktonic‐feeding larvae would actually be lower in nature. On the other hand, laboratory‐reared larvae are generally fed ad libitum, which might provide them with more food than they experience naturally, potentially growing faster. Despite these caveats, laboratory‐rearing trials for the intertidal barnacles *Amphibalanus amphitrite* (Anil et al., 2001; Darwin, 1854) and *Chthamalus proteus* Dando & Southward, 1980 (Zabin et al., 2007) varied both food concentrations and rearing temperature, and found little difference in PLD between food treatments, suggesting that food availability has little effect on PLD estimates. We therefore conclude that our results and conclusions are robust to the potential biases of laboratory approaches. Another bias relates to the underlying phylogenetic hypothesis, which solely reflects taxonomy where phylogenetic information is lacking. This means the tree has many polytomies, which could hamper accurate trait reconstruction. For our analyses, this might not have played a large role because trait transitions were infrequent, and closely related species generally shared similar traits. Lastly, sampling biases might have led to underestimates for particular traits. Particular deep sea and Antarctic regions are much less explored than shallow coastal waters of temperate and tropical regions. We show that brooded and planktonic‐nonfeeding larvae are more common in these regions. Further explorations should identify more species, which could shift our relative trait abundance.

Phylogenetic conservatism in barnacles has previously been suggested for larval developmental mode (Barnard, 1924) and egg size (Barnes & Barnes, 1965), which is confirmed by the strong phylogenetic signal. Such a significant phylogenetic signal can be caused by developmental or genetic constraints, a low rate of evolutionary change, strong stabilizing selection, epistatic interactions with other traits, or adaptive radiation into similar niches (Harvey & Purvis, 1991; Losos, 2008; Revell, Harmon, & Collar, 2008; Wiens & Graham, 2005). Egg size, larval size, and larval developmental mode show signs of adaptive evolution because these traits, correlated with temperature, water depth, or chlorophyll *a* concentration (see e.g., Ibáñez et al., 2018 for similar conclusions). The combination of both strong phylogenetic and adaptive signals could be the result of phylogenetic niche conservatism, possibly caused by adaptive radiations of closely related species into similar niches (Ackerly, 2009; Harvey & Purvis, 1991).

Future comparative studies should investigate the sister taxon to the Thoracica, the Rhizocephala. The Rhizocephala are particularly interesting because all species have nonfeeding larvae yet they are very small; their size never exceeds 300 µm, and larvae are much smaller in some species. Moreover, rhizocephalan larvae do not appear to be especially thick and loaded with yolk. Obviously, these parasites are also facing the dilemma between lecithotrophy and producing enormous numbers of offspring to successfully invade a host, which they might have solved in a unique way.

## 5 CONCLUSION

The results of our phylogenetic analyses on the life history of barnacle larvae are congruent with two adaptive hypotheses explaining the distribution of larval life history traits. Firstly, the distribution of larval developmental modes corresponds with water temperature and water depth, which is consistent with the predictions of the plankton‐duration hypothesis and Thorson's rule, but our data do not support the assumption that planktonic larval duration is affected by egg size. Secondly, egg size is closely correlated with larval size, and the latter correlates with water temperature, which agrees with Strathmann's size advantage hypothesis. Thirdly, the presence of a planktonic phase is correlated with phytoplankton abundance, which is also the case in other marine invertebrates (Marshall et al., 2012), but lacks an adaptive explanation at this point. Only planktonic larval duration (PLD) did not correlate with any of the environmental variables, thus lacking an adaptive signal based on our analyses. We surmise that PLD could still have evolved adaptively, but that we did not analyse the appropriate selective forces. In summary, we could not agree more with Strathmann (1977), who concluded 40 years ago that hypotheses “outlining ‘adaptive strategies’ present a much clearer picture than we can obtain from Mother Nature, who allows multiple functions for multiple interrelated traits and allows accidents of ancestry to place limits on adaptive variation.”

## CONFLICT OF INTEREST

None declared.

## AUTHOR CONTRIBUTIONS

Both authors collected life history data from the literature. PP extracted geographic occurrences and extrapolated environmental data. CE‐S carried out the statistical comparative analyses. Both authors contributed to manuscript writing and approved the final draft.

## Data Availability

We deposited life history and summary environmental data in https://datadryad.org (https://doi.org/10.5061/dryad.s8800t9). We used the synthesis phylogeny for barnacles of Ewers‐Saucedo et al. (2019) to account for phylogenetic nonindependence. The editable tree file (Appendix S10) is a supplement of their publication in peerJ.
