# Supplementary material for: Testing adaptive hypotheses on the evolution of larval life history in acorn and stalked barnacles
Source: Ecol Evol. 2019 Sep 18;9(19):11434–47. doi: 10.1002/ece3.5645 (PMC6802071; doi:10.1002/ece3.5645)
Supplement: Supplementary file 1 [file ECE3-9-11434-s001.pdf]

Supplement S1: Relationship between egg size and size of the first nauplius larvae in free-living barnacles (Thoracica)

From C. Ewers-Saucedo & P. Pappalardo "Evidence for adaptive phylogenetic niche conservatism in the larval development of marine invertebrates"

Objective

We wanted to use egg length as our measure of offspring size. However, egg size was not always reported. Instead, the length of the first nauplius larvae (the larvae that hatches from the egg) was reported in several instances. These two measures should be closely related, and we wanted to formally quantify this relationship.

Material and methods

To assess if the length of the first nauplius larvae is a good predictor of egg length, we modeled their relationship with a linear regression using data from all species for which both measurements were taken. We then used the fitted model to convert naupliar length to egg length for any species for which egg length was not reported.

Results

Data on both egg length and larval length were available for 64 species. A linear regression provided a good fit between the two variables (p-value < 0.0001, R-squared = 0.817). The intercept was -9.749, and the slope 0.910, suggesting that eggs are somewhat smaller than larvae (Fig. S1).

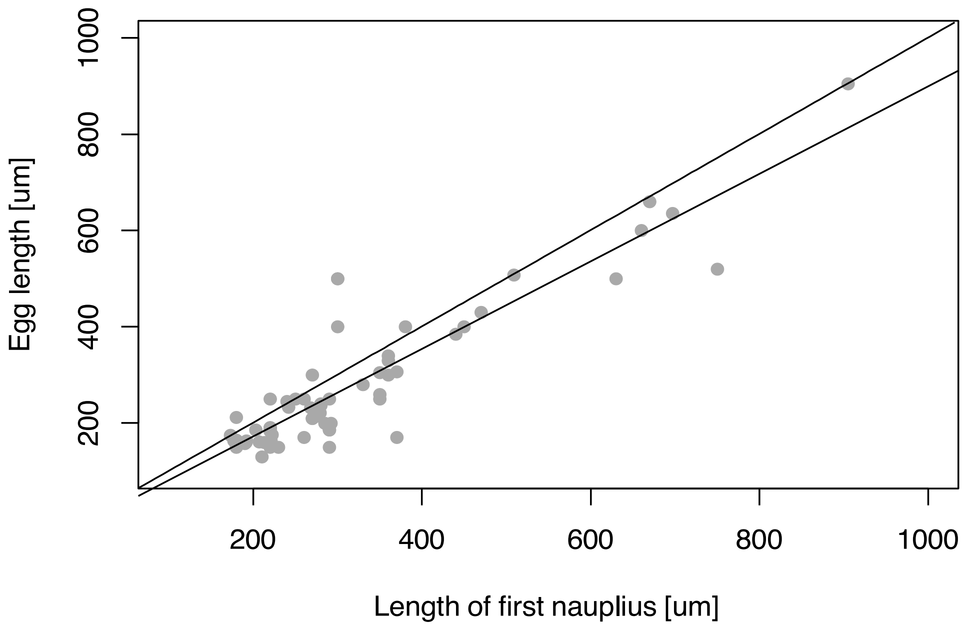

Figure S1. Relationship between egg length and length of the first nauplius larvae. Solid line denotes the relationship predicted by the linear model, the broken line denotes the one-to-one relationship.
