# Supplementary material for: Testing adaptive hypotheses on the evolution of larval life history in acorn and stalked barnacles
Source: Ecol Evol. 2019 Sep 18;9(19):11434–47. doi: 10.1002/ece3.5645 (PMC6802071; doi:10.1002/ece3.5645)
Supplement: Supplementary file 2 [file ECE3-9-11434-s002.pdf]

Supplement S2: Calculation of temperature-dependent Q10 to normalize PLD

From: C. Ewers-Saucedo & P. Pappalardo “Evidence for adaptive phylogenetic niche conservatism in the larval development of marine invertebrates”

Objective

Temperature has a large effect on the development time of marine larvae (O'Connor et al. 2007), and thus larvae reared under different temperatures will differ in planktonic larval duration (PLD). PLD can be normalized to a common temperature (we chose 20°C) by applying the appropriate Q_{10} value. Q_{10} is generally comparable among different species of barnacles with values between 1.25 to 4.2 for developing eggs (Patel and Crisp 1960, Hines 1979), but increases with decreasing temperatures (Hoegh-Guldberg and Pearse 1995; Patel and Crisp 1960).

Material and Methods

In order to account for differences in rearing temperatures, we normalized PLD to 20°C as in Levitan (2000). We first calculated Q_{10} values for larval development. This was possible whenever larvae of the same species were reared under different temperatures using the following formula, which is equivalent to formula 12 of Levitan (2000):

$$Q_{10} = \frac{PLD_2}{PLD_1}^{\frac{10}{Z_2 - Z_1}},$$

where PLD_1 and PLD_2 are the development times at water temperatures Z_1 and Z_2, respectively. We tested whether Q_{10} changes with temperature by fitting a linear regression between the midpoint of rearing temperatures and the Q_{10} calculated from these temperatures, and tested for the significance of temperature and species with an ANOVA. We then normalized the experimental PLD by considering the Q_{10} value appropriate for the experimental temperature using again formula 12 of Levitan (2000):

$$PLD_{norm} = \frac{1}{Q_{10}}^{\frac{Z_{norm} - Z_{exp}}{10}} + PLD_{exp},$$

where PLD_{exp} and Z_{exp} were the experimental PLD and water temperature, respectively. Z_{norm} was 20°C. PLD_{norm} is the normalized PLD at 20°C used in all further analyses. For species with more than one PLD estimate per experiment, we calculated the mean normalized PLD. We compared the standard deviation of all normalized PLD values of an experiment with the standard deviation of PLD values obtained at different temperatures in the same experiment using an one-sided paired t-test. If normalization to a single temperature makes PLD values more similar, we expect the standard deviation for normalized PLD to be lower than the original PLD values.

Results

In 16 species, PLD was estimated in the same experimental setup at different temperatures (same laboratory sensu Levitan 2000). Albeit some species developed slower than others, such as

Notochthamalus scabrosus (Fig. S2-1), the overall shape of the relationship between temperature and PLD was similar. Q_{10} ranged from 0.51 to 5.00 (median = 1.84, mean = 2.04). It decreased significantly with temperature (p-value = 0.023), but showed no significant species-specific effects (p-value = 0.6739) (Fig. S2-2). As expected, the standard deviations of normalized PLD values of the same species were significantly smaller than the standard deviation of original PLD values recorded from different temperatures (t = -2.72, df=17, p-value = 0.0072).

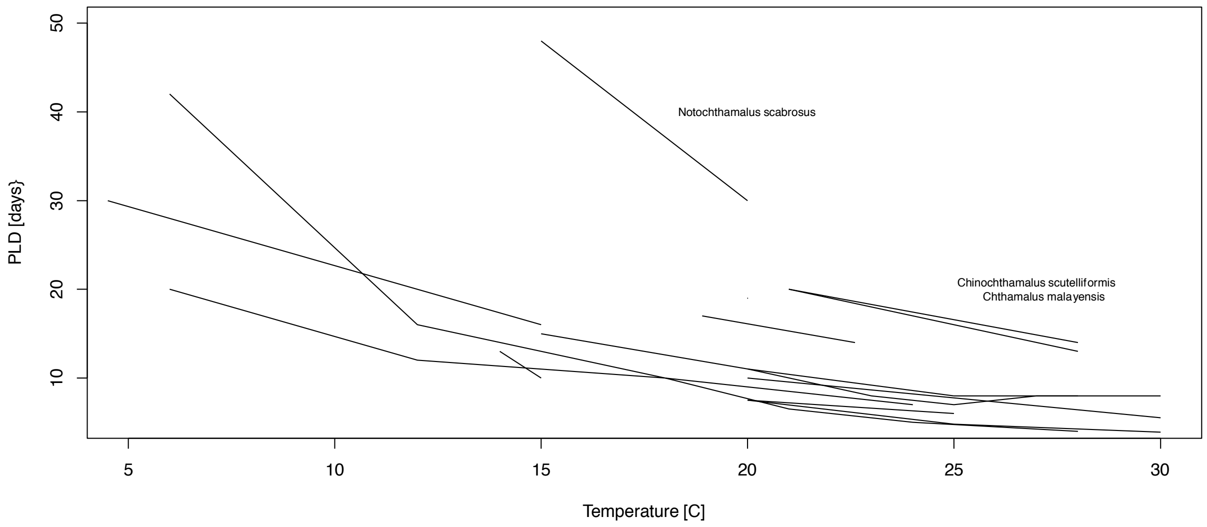

Figure S2-1. Temperature-dependent PLD estimates of different Thoracican species. Each line represents a rearing experiment, which reared larvae of the same species at different temperatures and under otherwise comparable conditions.

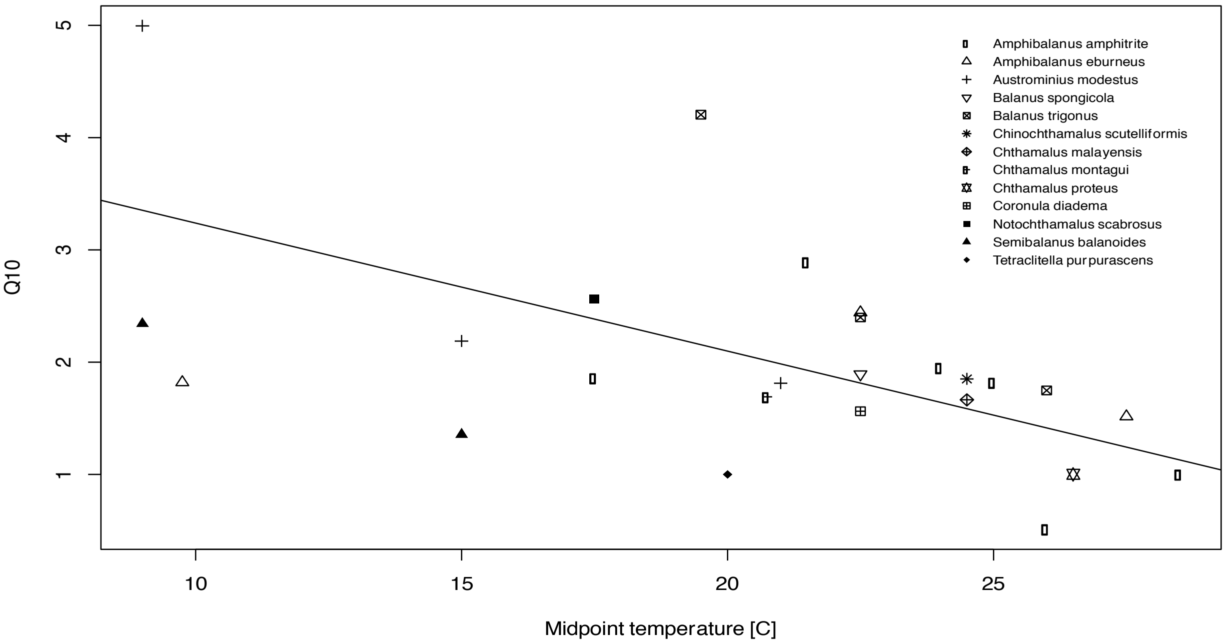

Figure S2-2. Relationship between Q_{10} and temperature, where the midpoint temperature is the average temperature of the two different rearing temperatures used to calculate Q_{10}.
