# Supplementary material for: Testing adaptive hypotheses on the evolution of larval life history in acorn and stalked barnacles
Source: Ecol Evol. 2019 Sep 18;9(19):11434–47. doi: 10.1002/ece3.5645 (PMC6802071; doi:10.1002/ece3.5645)
Supplement: Supplementary file 3 [file ECE3-9-11434-s003.pdf]

Supplement S3: Variation in offspring size and normalized PLD

From: C. Ewers-Saucedo & P. Pappalardo "Evidence for adaptive phylogenetic niche conservatism in the larval development of marine invertebrates"

Objective

Several studies report that populations of the same species have larger offspring at higher latitudes, which complicates the use of phylogenetically corrected analyses, as these rely on a single data point per species. Barnes and Barnes (1965), however, concluded that egg size did not change with latitude in most species. We aimed to confirm their observation statistically. We also assessed if planktonic larval duration (PLD) within species was correlated with latitude.

Material and Methods

We modeled a linear regression between egg size and latitude for each species for which egg sizes were measured at more than one location. The same approach was taken for normalized PLD and latitude.

Results

PLD was estimated at more than two locations in two species (Fig S3-1). The relationship between latitude and normalized PLD was significant for *Amphibalanus amphitrite* (p-value = 0.018), but not *Amphibalanus eburneus* (p-value = 0.018). In *A. amphitrite*, the significant result was due to an outlier PLD estimate from the southern hemisphere. The southern hemisphere estimate was much larger than the northern hemisphere estimates, and was removed in further analyses. The data at hand are too sparse to make any conclusions on the relationship between latitude and normalized PLD. Egg size was measured at more than two locations in eight species (Fig. S3-2). It did not vary significantly with latitude in any species. In most species, the variance between species was larger than the variance within species for both egg size and normalized PLD (Fig. S3). We therefore used the mean egg size +/- its standard error in all further analyses. For all species with only a single egg size or PLD measurement, we used the median standard error calculated from all species with more than one measurement.

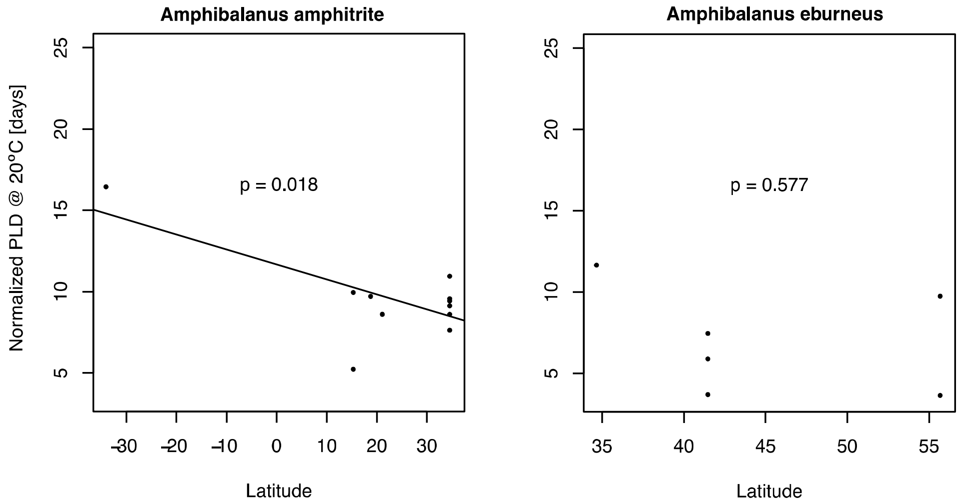

Figure S3-1. Relationship between latitude and PLD in species for which PLD was estimated at more than two locations.

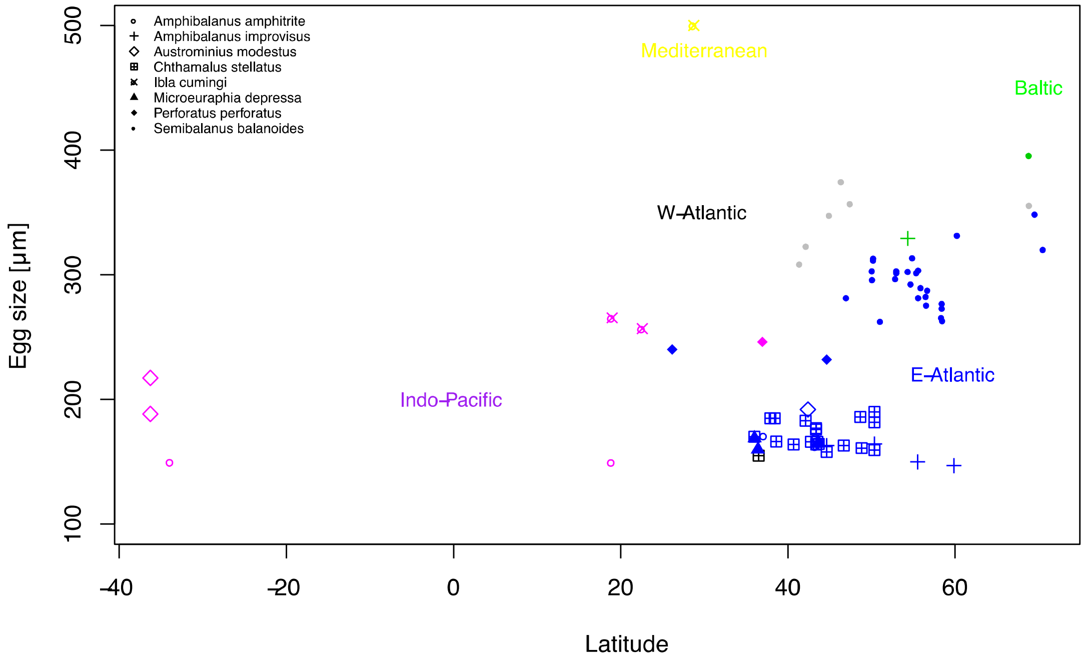

Figure S3-2. Relationship between latitude and egg size in species for which egg size was estimated at more than two locations.

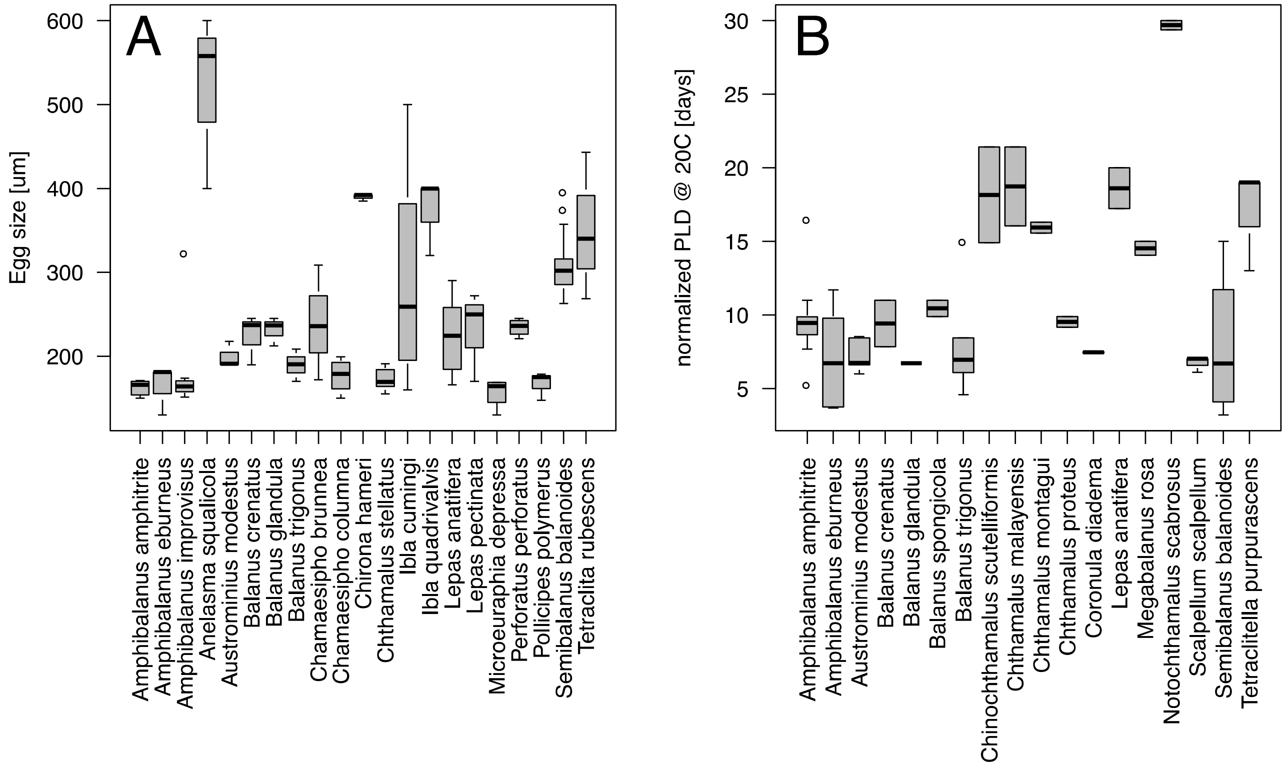

Figure S3-3. Species-specific variation in egg size (A) and normalized PLD (B).
