# Supplementary material for: Testing adaptive hypotheses on the evolution of larval life history in acorn and stalked barnacles
Source: Ecol Evol. 2019 Sep 18;9(19):11434–47. doi: 10.1002/ece3.5645 (PMC6802071; doi:10.1002/ece3.5645)
Supplement: Supplementary file 4 [file ECE3-9-11434-s004.pdf]

Supplement S4: How to combine barnacle occurrences and extract environmental data

From: C. Ewers-Saucedo & P. Pappalardo "Evidence for adaptive phylogenetic niche conservatism in the larval development of marine invertebrates"

Objective

Finding occurrence points for the barnacle species present in our life history database to infer depth distribution of species and extract environmental data associated to each point.

Materials and methods

Occurrence data. We extracted occurrence data from 1) the Ocean Biogeographic Information System (OBIS: http://www.iobis.org, consulted January 2017), 2) from the Global Biodiversity Information Facility (GBIF: http://www.gbif.org, consulted January 2017) and 3) from collection points reported in the literature (collected by us up to January 2017). As a first filter, we extracted data from OBIS using as key words "Cirripedia" and the "point" downloading option to get the full information (with the check for *data as provided by source*); data from GBIF was selected with taxonomic filter set as "Sessilia or Iblidae or Pedunculata or Akentrogonida or Dendrogastrida or Pygophora or Cyprilepadidae Newman, Zullo & Withers, 1969 or Apygophora or Scalpelliformes or Lithoglyptidae or Lepadidae or Kentrogonida"; and downloaded using the *Darwin Core Archive* option that provides all details available. We searched for occurrence data using the taxonomic names published in the Open Tree of Life, as well as their synonyms. For synonyms, we followed the World Register of Marine Species (WoRMS Editorial Board 2015) and ITIS (Integrated Taxonomic Information System: www.itis.gov/). We incorporated all occurrences for the species included in our database and their synonyms, using only the currently accepted species name in subsequent manipulations. All the data manipulation was done in R (R Core Team 2016).

From the general output we kept only the occurrences that correspond to the species present in our life history database and that had information on latitude and longitude or on depth. Additionally, we added geographic coordinates to the literature occurrence when locality was reported in the original source. When depth was available for an occurrence point we recorded depth. Often in the literature we found depth distribution of species reported as minimum and maximum, in those cases we recorded both values. In the OBIS data, the "depth" column is highly correlated with the "woa_depth" column ($R2= 0.997$); we averaged both values. In GBIF, there were instances where the "depth" column was empty, but the "verbatim_depth" column had non numeric information on depth. To use that information, we converted the information to numeric when it indicated "30m" for example. When depth was indicated in fathoms (example "30ft"), we converted it to meters. For the records "surface", "shore", "intertidal" we indicated a depth of 0 meters.

We merged the occurrences from the three sources in a combined dataset. We estimated the median depth for each species as the median of all the combined depth information from OBIS, GBIF and the information from the literature. Because we were not interested in the proportion of occurrences at a point, we deleted entries that were duplicated in all columns (not considering the institution code column). Since is not uncommon to find some errors when using big databases, we

double-checked that the occurrences were not mapped to land. To filter occurrences falling on land we used the object "wrld_simpl" from the *maptools* package (Bivand and Lewin-Koh 2016) as our reference for land and with the function "over" in the *sp* (Pebesma and Bivand 2005, Bivand et al 2013) package we were able to check what points intercepted land. Most of the points in land are coastal points, probably belonging to intertidal species in which the collection point was a locality on the coast. If the occurrence was georeferenced as the midpoint of a coastal locality, the midpoint is likely to fall in land. The approach we used to interpolate the oceanographic variables uses the nearest point in the oceanographic grid. By checking that the distances were really small between the occurrence point and the grid we were able to use the nearshore "land" points, but avoiding occurrences in the middle of continents that may be errors (or museum specimens where the location of the museum was the georeferenced point).

Environmental data. We searched for information on ocean temperature, chlorophyll a concentration (a proxy for food availability), and water depth for each occurrence in our database. We manipulated, summarized and analyzed the oceanographic data in R (R Development Core Team 2016) using the packages "ncdf4" version 1.15 (Pierce 2014), "RColorBrewer" v. 1.1-2 (Neuwirth 2014), "lattice" v. 0.20-34 (Sarkar 2008), "fields" v. 8.10 (Nychka et al. 2015), "plyr" v. 1.8.4 (Wickham 2011) and "RANN" v. 2.5 (Arya 2015). We considered spring data in our analysis of environmental variables because usually larval release of marine invertebrates occurs in spring and early summer (Hines 1979; Barnes 1989; Anderson 1994; Byers and Pringle 2006). Depending on the platform we used to extract data, there were slight differences in how spring is defined (details below for each variable), but we divided our data to consider the northern hemisphere spring and the southern hemisphere spring. To interpolate the gridded oceanographic data to our occurrences we used the function "nn2" in the "RANN" package. From all the occurrences for each species we calculated the median value of each environmental variable and the interquartile range (IQR) to represent variation. When we could not compute the interquartile range due to insufficient data, we used the overall median of interquartile ranges from all species with this information.

To classify species by depth distribution, we combined the depth range reported in the literature with depth information available for the OBIS/GBIF occurrences. We calculated median depth and interquartile range (IQR) to represent a species' depth distribution. When only one observation was available, we used the median IQR calculated from all species for which we could calculate IQR. We defined shallow species as those with a median depth less than 30m, which was the subset of species included in the chlorophyll *a* analysis. Some species of neustonic barnacles (those living in floating substrate) were mis-classified as deep sea species based in OBIS and GBIF. To ensure they are classified as shallow-water species, we assigned a minimum depth of 0, a maximum depth of 30m and a median depth of 5m to Lepas pectinata, L. australis, L. anatifera, L. anserifera and Dosima fascicularis.

In situ seasonal temperature was downloaded as a NetCDF file from the World Ocean Atlas 2013 database (http://www.nodc.noaa.gov/cgi-bin/OC5/woa13/woa13.pl), with a resolution of 1/4°. We selected the objectively analyzed climatology of seawater temperature for spring in both hemispheres, for the time period 2005-2012. In the World Ocean Atlas data, spring for the northern hemisphere is defined as Apr-May-Jun, and spring for the southern hemisphere (corresponding to

autumn files) defined as Oct-Nov-Dec. Seawater temperature data are generally available for standard depth levels between 0m and 5500m, which allowed us to interpolate temperature values for occurrences in each layer of depth. This database contains considerable amounts of missing data, and we took two approaches. The first approach was to estimate the "exact" temperature of each occurrence point based on that occurrence depth; subsequently, values of temperature were averaged for each species. With this method, we were able to estimate mean temperature for only 122 species, due to missing information on the interpolated grid. The second approach was to average the temperature values in the two layer depths that included the median depth of the species; this approach resulted in temperature estimates for 150 species. Both measures were highly correlated (Pearson correlation: R-squared = 0.93, p-value < 0.0001), and we used the second approach to calculate median temperature and interquartile range (IQR). When only one observation was available, we used the median IQR calculated from all species for which we could calculate IQR.

SeaWiFS (spatial resolution: 9km) estimates of chlorophyll a concentration were downloaded as NetCDF file from the Giovanni online data system (https://giovanni.sci.gsfc.nasa.gov/giovanni/), developed and maintained by the NASA GES DISC. We selected the user-defined climatology options to download seasonal data; in the Giovanni system, the seasons are defined slightly different. We used March-April-May (northern hemisphere spring) and September-October-November (southern hemisphere spring), for the time period 2005-2012 to match the temperature data. Based on this data, we interpolated the chlorophyll a concentration for most of the occurrences of the shallow water species in our database. Chlorophyll a was log transformed for all analyses. We calculated the median and interquartile range (IQR) of the log-transformed chlorophyll. When only one observation was available, we used the median IQR calculated from species for which we could calculate IQR. Chlorophyll a concentration was used as proxy for food availability in shallow water species (< 30m). Remote sensing data integrates chlorophyll a concentration across water layers, whereby the maximal depth depends on water properties such as visibility, and commonly ranges from 20 to 200m (Lorenzen 1970; Campbell and O'Reilly 1988; Schalles 2006). Using only shallow water species (median depth < 30m) is therefore a conservative estimate.

Results

Below we present all the occurrences collected for this study, showing in black dots the occurrences that mapped correctly in the ocean, and in red dots the occurrences that appear to be in land.

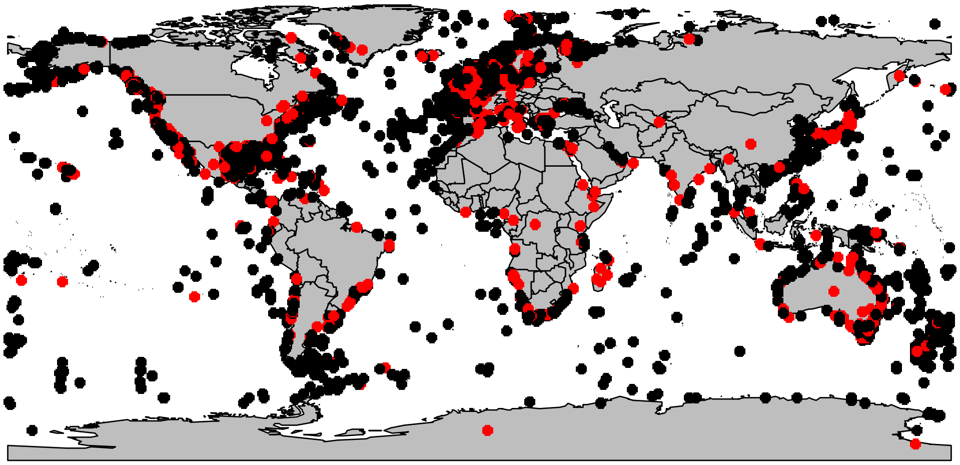

References

Anderson, D. T. 1994. Barnacles: Structure, Function, Development and Evolution. Chapman & Hall, London, UK.

Arya, S. 2015. RANN: Fast nearest neighbor search.

Bivand R. and N. Lewin-Koh (2016). maptools: Tools for Reading and Handling Spatial Objects. R package version 0.8-39. https://CRAN.R-project.org/package=maptools

Bivand R., E. Pebesma, and V. Gomez-Rubio (2013). Applied spatial data analysis with R, Second edition. Springer, NY. http://www.asdar-book.org/

Byers, J. E., and J. M. Pringle. 2006. Going against the flow: retention, range limits and invasions in advective environments. Marine Ecology Progress Series 313:27–41.

Campbell, J. W., and J. E. O'Reilly. 1988. Role of satellites in estimating primary productivity on the northwest Atlantic continental shelf. Continental Shelf Research 8:179–204.

Hines, A. H. 1979. The Comparative Reproduction Ecology of Three Species of Intertidal Barnacles.

Lorenzen, C. J. 1970. Surface chlorophyll as an index of the depth, chlorophyll content, and primary productivity of the euphotic layer. Limnology and Oceanography 15:479–480.

Neuwirth, E. (2014). RColorBrewer: ColorBrewer Palettes. R package version 1.1-2. https://CRAN.R-project.org/package=RColorBrewer

Nychka, D., R. Furrer, and S. Sain. 2015. fields: Tools for Spatial Data. R package version 8.2-1.

Pebesma, E.J. and R.S. Bivand (2005). Classes and methods for spatial data in R. R News 5 (2), http://cran.r-project.org/doc/Rnews/.

Pierce, D. 2014. ncdf4: Interface to Unidata netCDF (version 4 or earlier) format data files.

R Core Team (2016). R: A language and environment for statistical computing. R Foundation for Statistical Computing, Vienna, Austria. URL https://www.R-project.org/.

Sarkar, D. 2008. Lattice: multivariate data visualization with R. Springer Science & Business Media.

Schalles, J. F. 2006. Optical remote sensing techniques to estimate phytoplankton chlorophyll a concentrations in coastal waters with varying suspended matter and CDOM conentrations. Pp. 27–79 in L. L. Richardson and E. F. LeDrew, eds. Remote sensing of aquatic coastal ecosystem processes. Springer Netherlands, Dordrecht.
