# Supplementary material for: Testing adaptive hypotheses on the evolution of larval life history in acorn and stalked barnacles
Source: Ecol Evol. 2019 Sep 18;9(19):11434–47. doi: 10.1002/ece3.5645 (PMC6802071; doi:10.1002/ece3.5645)
Supplement: Supplementary file 5 [file ECE3-9-11434-s005.pdf]

Supplement S5: Testing a non-linear relationship between egg size and PLD

From: C. Ewers-Saucedo & P. Pappalardo "Evidence for adaptive phylogenetic niche conservatism in the larval development of marine invertebrates"

Objective

Levitan (2000) proposed that PLD is proportional to egg volume rather than linearly related to egg diameter. We tested this relationship for thoracican, taking the non-independence of species-trait data into account.

Materials and methods

We calculated the egg volume S for each species as the volume of an ovoid, based on length and width egg measurements from our literature search. We calculated "T" from equation (7) of Levitan (2000) for each species as:

$$T = (S_{fp} / S - 1) + PLD_{fp},$$

where S_{fp} the minimal egg volume of species with planktonic-feeding larvae and PLD_{fp} is its PLD. We used the smallest egg volume of species with planktonic-feeding larvae available in our data set, 0.00011μm^3 for *Chelonibia testudinaria*, and its respective PLD of 17 days (Zardus & Hadfield 2004). If Levitan's model holds for barnacles, "T" should approximate PLD for each species, and these two variables should therefore be linearly correlated. To test this prediction, we fitted phylogenetic generalized least squares (PGLS) models between egg volume and Levitan's T while taking larval mode into account. We modeled covariance based on the best-fit model of trait evolution (see main manuscript section: "Multiple model comparison and ancestral state reconstruction") and took trait variation into account (function "gls", R package "nlme").

Results

When accounting for larval mode, egg volume was not correlated to Levitan's "T" (p-value = 0.264), suggesting that egg volume does not scale proportionally with PLD.

References

Levitan, D. R. 2000. Optimal Egg Size in Marine Invertebrates: Theory and Phylogenetic Analysis of the Critical Relationship between Egg Size and Development Time in Echinoids. The American Naturalist 156:175–192.

Zardus, J. D., and M. G. Hadfield. 2004. Larval Development and Complemental Males in *Chelonibia testudinaria*, a Barnacle Commensal with Sea Turtles. Journal of Crustacean Biology 24:409–421.
