# Supplementary material for: Testing adaptive hypotheses on the evolution of larval life history in acorn and stalked barnacles
Source: Ecol Evol. 2019 Sep 18;9(19):11434–47. doi: 10.1002/ece3.5645 (PMC6802071; doi:10.1002/ece3.5645)
Supplement: Supplementary file 6 [file ECE3-9-11434-s006.pdf]

**Supplement S6: Correlations between environmental variables**

From: C. Ewers-Saucedo & P. Pappalardo "Evidence for adaptive phylogenetic niche conservatism in the larval development of marine invertebrates"

## Objective

One of the overall aims of our study was to correlate larval traits with environmental variables. As using several tightly-correlated variables within the same model can lead to erratic model outcomes, also known as collinearity, we assessed how correlated the environmental variables were to each other. The environmental variables were water depth, temperature and chlorophyll *a* concentration. See the main manuscript and Supplement S4 for details on data acquisition.

## Material and methods

Water depth and chlorophyll a concentration were log-transformed prior to analyses. We used linear regressions on pairwise environmental variables and Pearson's product-moment correlation on all pairs of environmental variables. For chlorophyll *a* concentration, we only considered data from less than 20m water depths, which ensured that we only considered the photic zone. We did not correlate chlorophyll *a* concentration against water depth because the chlorophyll concentration represents an integrated estimate across the photic zone, and it would therefore be inappropriate to correlate it with a specific depth estimate within the photic zone. ~~See the main manuscript for details on data collection.~~

## Results

The most significant linear relationship existed between water depth and temperature (R-squared = 0.54, p-value < 0.001), whereby temperatures were most variable on the surface (Fig. S6A). Chlorophyll *a* concentration and temperature were also well-correlated (R-squared = 0.46, p-value < 0.001) (Fig. S6B). Similar results were obtained for Pearson's correlation coefficients: Water depth and temperature are tightly correlated (Pearson's product-moment correlation coefficient = 0.74, p-value < 0.001), as are temperature and chlorophyll *a* concentration (Pearson's product-moment correlation coefficient = 0.69, p-value < 0.001). These variables should therefore not be considered in the same model.

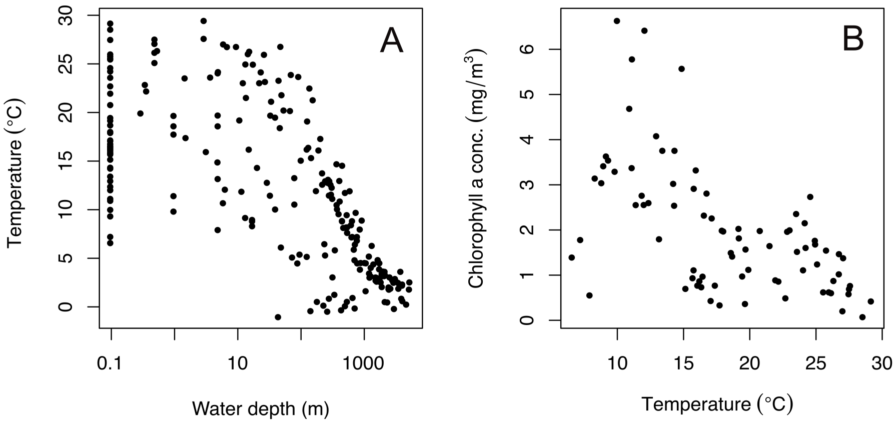


Figure S6. Relationships between environmental variables.
