# Supplementary material for: Testing adaptive hypotheses on the evolution of larval life history in acorn and stalked barnacles
Source: Ecol Evol. 2019 Sep 18;9(19):11434–47. doi: 10.1002/ece3.5645 (PMC6802071; doi:10.1002/ece3.5645)
Supplement: Supplementary file 7 [file ECE3-9-11434-s007.pdf]

Supplement S7: Models of trait evolution

Objective

Models of trait evolution describe how traits evolve along a phylogenetic tree. Given a specific set of characters, some models are more successful at explaining the distribution of traits than others. The best-fitting model should be used to account for the non-independence of species in comparative methods. A number of models of trait evolution can be interpreted as transforming the phylogenetic tree in specific ways: Brownian Motion (BM) is modeled on an untransformed tree (Felsenstein 1985), the absence of phylogenetic signal is modeled on a star-like tree, and the extent to which phylogeny predicts covariance among trait values is modeled on a lambda-transformed tree (Pagel 1999). The lambda-model also estimates the most likely value of lambda, which provides additional insight into the evolutionary process. If lambda equals zero, phylogenetic signal is absent, and the phylogenetic tree is star-like, while a lambda value of one represents neutral trait evolution under BM (Pagel 1999; Freckleton et al. 2002). Intermediate values of lambda suggest that, although the trait has a phylogenetic signal, it has not evolved under a pure BM process (Pagel 1999; Freckleton et al. 2002). A delta transformation of the tree models time-dependent trait evolution (Pagel 1999). A delta value smaller than one means that recent evolution has been slow, while a delta value larger than one indicates relatively fast recent evolution. The Ornstein-Uhlenbeck model, an extension of the BM model applicable to continuous traits, models the evolution of traits towards an adaptive optimum (Butler & King 2004). The strength of selection towards this optimum is modeled by the parameter alpha, where an alpha of zero collapses the model to BM. The objective was to identify the best-fitting model of trait evolution for thoracican barnacle larval traits based on our data.

Materials and methods

We fit different models of trait evolution to our data: Brownian Motion (BM), lambda or delta tree transformations, and the Ornstein-Uhlenbeck model. Then we selected the best-fitting model using AIC weights (Wagenmakers and Farrell 2004) For the continuous traits egg size and PLD, we used the function “fitContinuous” in the R package “geiger”. For larval developmental mode

with its three discrete trait states, we used the function "fitDiscrete" in the R package "geiger". For such discrete traits, transition rates between modes of larval development can either vary, or be the same. It is also possible to allow some rates to vary, while others are constrained to be the same, but we restricted our analyses to the two extreme cases: either all rates differ, or all rates are the same.

Results

The evolution of larval mode (planktonic-feeding, planktonic-nonfeeding, non-planktonic) was best modeled under a lambda-transformed phylogeny with a lambda model parameter of 0.81 and equal transition rates (Table S7-1A). Egg size and PLD were also best modeled under lambda-transformed trees with lambdas of 0.788 and 0.884, respectively (Table **S7-1**B,C). These results suggest that all larval traits have a significant phylogenetic signal, as indicated by the high lambda value, but that they did not evolve under BM (Pagel 1999; Freckleton et al. 2002). We used the best-fitting lambda models to account for the non-independence of species-level trait data in the ancestral state reconstruction and all of the PGLS correlations performed in the main manuscript.

Table S7-1. Multiple model selection for models of larval trait evolution of thoracican barnacles.

		Model	Transition rates	AIC	ΔAIC	AIC weights
A	Larval mode	Lambda	equal	147.790	0.000	0.788
		Lambda	different	154.589	6.799	0.026
		BM	different	155.206	7.417	0.019
		BM	equal	158.122	10.332	0.004
		No phylo. signal	equal	265.884	118.094	0
		No phylo. signal	different	265.884	118.094	0
B	Egg size	Lambda	NA	1800.475	0.000	0.999
		OU	NA	1815.069	14.594	0.001
		Delta	NA	1818.025	17.55	0
		BM	NA	1827.687	27.212	0
		No phylo. signal	NA	1904.999	104.524	0
C	PLD	Lambda	NA	623.345	0.000	1.000
		OU	NA	648.552	25.207	0
		Delta	NA	651.386	28.041	0
		BM	NA	76338.641	75715.296	0
		No phylo. signal	NA	76332.798	75709.453	0

(A) Larval mode: the three larval developmental modes planktonic-feeding, planktonic-nonfeeding and non-planktonic (see fig. 1 of the main text). (B) Egg size, measured as maximal egg diameter in µm. (C) PLD, the planktonic larval duration, measured in days. We compared the fit of the following models to our data: BM = Brownian Motion (Felsenstein 1973); lambda = model based on Pagel's (1999) lambda;

delta = model based on Pagel's (1999) delta; OU = Ornstein-Uhlenbeck model (Butler et al. 2004); No phylo. signal = models fit of the data without phylogenetic information. Abbreviations: AIC = Akaike Information Criterion; PLD = planktonic larval duration.

References

Butler, M. A., A. A. King, and B. J. Crespi. 2004. Phylogenetic comparative analysis: a modeling approach for adaptive evolution. The American Naturalist 164:683–695.

Felsenstein, J. 1985. Phylogenies and the comparative method. American Naturalist 1–15.

Freckleton, R. P., P. H. Harvey, and M. Pagel. 2002. Phylogenetic analysis and comparative data: a test and review of evidence. The American Naturalist 160:712–726.

Pagel, M. 1999. Inferring the historical patterns of biological evolution. Nature 401:877–884.

Wagenmakers, E.-J., and S. Farrell. 2004. AIC model selection using Akaike weights. Psychonomic Bulletin & Review 11:192–196.
