# Supplementary material for: Testing adaptive hypotheses on the evolution of larval life history in acorn and stalked barnacles
Source: Ecol Evol. 2019 Sep 18;9(19):11434–47. doi: 10.1002/ece3.5645 (PMC6802071; doi:10.1002/ece3.5645)
Supplement: Supplementary file 9 [file ECE3-9-11434-s009.rtf]

Achituv, Y. 1986. The Larval Development of Chthamalus dentatus Krauss (Cirripedia) from South Africa. Crustaceana 51:259–69.Anderson, D. T. 1994. Barnacles: Structure, Function, Development and Evolution. Chapman & Hall, London, UK.Anderson, D. T. 1965. Embryonic and larval development and segment formation in Ibla quadrivalvis Cuv. (Cirripedia). Australian Journal of Zoology 13:1–5.Anderson, D. T. 1986. The circumtropical barnacle Tetraclitella divisa (Nilsson-Cantell) (Balanomorpha, Tetraclitidae): cirral activity and larval development.Anderson, D. T., J. T. Anderson, and E. A. Egan. 1988. Balanoid barnacles of the genus Hexaminius (Archaeobalanidae: Elminiinae) from mangroves of New South Wales, including a description of a new species. Records of the Australian Museum 40:205–23.Anil, A. C., D. Desai, and L. Khandeparker. 2001. Larval development and metamorphosis in Balanus amphitrite Darwin (Cirripedia; Thoracica): significance of food concentration, temperature and nucleic acids. Journal of Experimental Marine Biology and Ecology 263:125–41.Aurivillius, C. W. S. 1894. Neue Cirripedien aus dem Atlantischen, Indischen und Stillen Ocean. Övers. Kongl. Vetensk. Akad. Föh., Stockholm, Sweden.Barker, M. F. 1976. Culture and morphology of some New Zealand barnacles (Crustacea: Cirripedia). New Zealand Journal of Marine and Freshwater Research 10:139–58.Barnard, K. H. 1924. Contributions to the Crustacean Fauna of South Africa. 7. Cirripedia. Annals of The South African Museum 20.Barnes, H., and M. Barnes. 1965. Egg Size, Nauplius Size, and Their Variation with Local, Geographical, and Specific Factors in Some Common Cirripedes. Journal of Animal Ecology 34:391–02.Barnes, H., and J. D. Costlow. 1961. The larval stages of Balanus balanus (L.) Da Costa. Journal of the Marine Biological Association of the United Kingdom 41:59–8.Barnes, M. 1989. Egg production in cirripedes. Oceanographic Marine Biology: An Annual Review 27:91–66.Barnes, M., and Y. Achituv. 1981. The nauplius stages of the cirripede Tetraclita squamosa rufotincta Pilsbry. Journal of Experimental Marine Biological Ecology 54:149–65.Bassindale, R. 1936. The Developmental Stages of Three English Barnacles, Balanus balanoides (Linn.), Chthamalus stellatus (Poli), and Verruca stroemia (O. F. Müler). Proceedings of the Zoological Society of London 106:57–4.Batham, E. J. 1945a. Description of female, male and larval forms of a tiny stalked barnacle, Ibla idiotica. Transactions of the Royal Society of New Zealand 75:347–56.Batham, E. J. 1945b. Pollicipes spinosus Quoy and Gaimard. II. Embryonic and larval development. Transactions of the Royal Society of New Zealand 75:405–18.Biccard, A. 2012. Taxonomy, systematics and biogeography of South African Cirripedia (Thoracica). University of Cape Town.Branscomb, E. S., and K. Vedder. 1982a. A Description of the Naupliar Stages of the Barnacles Balanus glandula Darwin, Balanus cariosus Pallas, and Balanus crenatus Bruguièe (Cirripedia, Thoracica). Crustaceana 42:83–5.Branscomb, E. S., and K. Vedder. 1982b. A description of the naupliar stages of the barnacles Balanus glandula Darwin, Balanus cariousus Pallas, and Balanus crenatus Brugiere (Cirripedia, Thoracica). Crustaceana 42:83–5.Brickner, I., Y. Loya, and Y. Achituv. 2010. Diverse life strategies in two coral-inhabiting barnacles (Pyrgomatidae) occupying the same host (Cyphastrea chalcidicum), in the northern Gulf of Eilat. Journal of Experimental Marine Biology and Ecology 392:220–27.Brown, S. K., and J. Roughgarden. 1985. Growth, morphology, and laboratory culture of larvae of Balanus glandula (Cirripedia: Thoracica). Journal of Crustacean Biology 5:574–90.Brown, T. 1844. Illustrations of the recent conchology of Great Britain and Ireland, with the description and localities of all the species, marine, land, and fresh water. 2d ed., greatly enl. Smith, Elder, and Co.; [etc., etc.], London,.Buckeridge, J. S., and W. A. Newman. 2006. A revision of the Iblidae and the pedunculate barnacles (Crustacea: Cirripedia: Thoracica), including new ordinal, familial and generic taxa, and two new species from New Zealand and Tasmanian waters. Zootaxa 1136:1–8.Buhl-Mortensen, L., and J. Høg. 2006. Reproduction and larval development in three scalpellid barnacles, Scalpellum scalpellum (Linnaeus 1767), Ornatoscalpellum stroemii(M. Sars 1859) and Arcoscalpellum michelottianum (Seguenza 1876), Crustacea: Cirripedia: Thoracica): implications for reproduction and dispersal in the deep sea. Marine Biology 149:829–44.Buhl-Mortensen, L., and J. T. Høg. 2013. Reproductive strategy of two deep-sea scalpellid barnacles (Crustacea: Cirripedia: Thoracica) associated with decapods and pycnogonids and the first description of a penis in scalpellid dwarf males. Organisms Diversity & Evolution 13:545–57.Burrows, M. T., S. J. Hawkins, and A. J. Southward. 1999. A comparison of reproduction in co-occurring chthamalid barnacles, Chthamalus stellatus (Poli) and Chthamalus montagui Southward. Journal of Experimental Marine Biology and Ecology 160:229–49.Calcagno, J. A., and G. A. Lovrich. 2000. First Record of Notobalanus flosculus (Darwin, 1854) (Cirripedia, Archaeobalanidae) from the South Atlantic Coast of South America. Crustaceana 73:365–68.Campo, D., J. Molares, L. Garcia, P. Fernandez-Rueda, C. Garcia-Gonzalez, and E. Garcia-Vazquez. 2010. Phylogeography of the European stalked barnacle (Pollicipes pollicipes): identification of glacial refugia. Marine biology 157:147.Chan, B. K. K. 2009. Crustacean Fauna of Taiwan: Barnacles, Volume I - Cirripedia: Thoracica Excluding The Pyrgomatidae and Acastinae. National Taiwan Ocean Universtiy, Keelung, Taiwan.Chan, B. K. K. 2003. Studies on Tetraclita squamosa and Tetraclita japonica (Cirripedia: Thoracica) II: larval morphology. Journal of Crustacean Biology 23:522–47.Chan, B. K. K., and P. T. Y. Leung. 2007. Antennular morphology of the cypris larvae of the mangrove barnacle <span class="italic">Fistulobalanus albicostatus</span> (Cirripedia: Thoracica: Balanomorpha). Journal of the Marine Biological Association of the United Kingdom 87:913–15.Choi, K. H., K. H. Choi, D. T. Anderson, and C. H. Kim. 1992. Larval development of the megabalanine balanomorph Megabalanus rosa (Pilsbry) (Cirripedia, Balanidae). Proceedings of The Linnean Society of New South Wales 113:175–84.Coló-Urban, R., P. J. Cheung, G. D. Ruggieri, and R. F. Nigrelli. 1979. Observations on the development and maintenance of the deep sea barnacle, Octolasmis aymonini geryonophila (Pilsbry). International Journal of Invertebrate Reproduction 1:245–52.Costlow, J. D., and C. G. Bookhout. 1958. Larval development of Balanus eburneus in the laboratory. Biol. Bull. mar. biol. Lab., Woods Hole 114:284.Costlow Jr., J. D., and G. C. Bookhout. 1958. Larval Development of Balanus amphitrite Var. Denticulata Broch Reared in the Laboratory. Biological Bulletin 114:284–95.Crisp, D. J. 1959. Factors Influencing the Time of Breeding of Balanus balanoides. Oikos 10:275–89.Crisp, D. J. 1962. The Planktonic Stages of the Cirripedia Balanus balanoides (L.) and Balanus balanus (L.) from North Temperate Waters. Crustaceana 3:207–21.Dalley, R. 1984. The Larval Stages of the Oceanic, Pedunculate Barnacle Conchoderma Auritum (L.) (Cirripedia, Thoracica). Crustaceana 46:39–4.Daniel, A. 1972. Marine intertidal barnacles in the Indian Ocean. Marine survey division Zoological surveys of India.Darwin, C. 1851. A monograph on the sub-class Cirripedia, with figures of all the species. The Lepadidae, or, pedunculated cirripedes. Ray society, London.Dayton, P. K., W. A. Newman, and J. Oliver. 1982. The Vertical Zonation of the Deep-Sea Antarctic Acorn Barnacle, Bathylasma corolliforme (Hoek): Experimental Transplants from the Shelf Into Shallow Water. Journal of Biogeography 9:95–09.Denisenko, N. V., S. G. Denisenko, K. K. Lehtonen, A.-B. Andersin, and H. R. Sandler. 2007. Zoobenthos of the Cheshskaya Bay (southeastern Barents Sea): spatial distribution and community structure in relation to environmental factors. Polar Biology 30:735–46.Dineen, J. F. 1987. The larval stages of lithotrya dorsalis (ellis & solander, 1786): a burrowing thoracican barnacle. The Biological Bulletin 172:284–98.Egan, E. A., and D. T. Anderson. 1986. Larval Development of Balanus amphitrite Darwin and Balanus variegatus Darwin (Cirripedia, Balanidae) from New South Wales, Australia. Crustaceana 51:188–07.Egan, E. A., and D. T. Anderson. 1985. Larval development of Elminius covertus Foster and Hexaminius popeiana Foster (Cirripedia  Archaeobalanidae  Elminiinae) reared in the laboratory. Mar. Freshwater Res. 36:383–04.Egan, E. A., and D. T. Anderson. 1989. Larval development of the chthamaloid barnacles Catomerus polymerus Darwin, Chamaesipho tasmanica Foster & Anderson and Chthamalus antennatus Darwin (Crustacea: Cirripedia). Zoological Journal of the Linnean Society 95:1–8.Egan, E. A., and D. T. Anderson. 1988. Larval development of the coronuloid barnacles Austrobalanus imperator (Darwin), Tetraclitella purpurascens (Wood) and Tesseropora rosea (Krauss) (Cirripedia, Tetraclitidae). Journal of Natural History 22:1379–405.Ewers-Saucedo, C., J. M. Pringle, H. H. Sepúveda, J. E. Byers, S. A. Navarrete, and J. P. Wares. 2016. The oceanic concordance of phylogeography and biogeography: a case study in Notochthamalus. Ecology and Evolution 6:4403–420.Fabricius, K. E. 2006. Effects of irradiance, flow, and colony pigmentation on the temperature microenvironment around corals: Implications for coral bleaching? Limnology and Oceanography 51:30–7.Foster, B. A. 1989. Balanomorph barnacle larvae in the plankton at McMurdo Sound, Antarctica. Polar Biol 10:175–77.Foster, B. A. 1967. The early stages of some New Zealand shore barnacles. Tane 13:33–2.Gale, A. S. 2016. Phylogeny of the deep-sea cirripede family Scalpellidae (Crustacea, Thoracica) based on shell capitular plate morphology. Zoological Journal of the Linnean Society 176:266–04.Gannon, A. T. 1990. Distribution of Octolasmis muelleri: an ectocommensal gill barnacle on the blue crab. Bulletin  of Marine Science 46:55–1.Griffiths, R. J. I. 1979. The reproductive season and larval developmnt of the barnacle Tetraclita serrata Darwin. Transactions of the Royal Society of South Africa 44:97–11.Groom, T. T. 1894. On the early development of Cirripedia. Phil. Trans. R. Soc. B 185:1190232.Herrera, S., H. Watanabe, and T. M. Shank. 2014. Evolutionary and biogeographical patterns of barnacles from deep-sea hydrothermal vents. Molecular Ecology n/a-n/a.Herz, L. E. 1933. The Morphology of the Later Stages of Balanus Crenatus Bruguiere. Biological Bulletin 64:432–42.Hines, A. H. 1978. Reproduction in three species of intertidal barnacles from central California. Biol Bull 154:262–81.Hoek, P. P. C. 1883. Report on the Cirripedia collected by H.M.S. Challenger during the years 1873-76. Report of the Scientific Results from the Exploratory Voyages of H.M.S. Challenger, Zoology 8:1–69.Hoek, P. P. C. 1907. The Cirripedia of the Siboga Expedition. A. Cirripedia pedunculata. E.J. Brill, Leiden.Horning Jr, D. S. 1982. Littoral barnacles of The Snares islands, southern New Zealand (Cirripedia: Thoracica). New Zealand Journal of Zoology 9:319–23.Hosie, A. M. 2014. Review of the stalked barnacle genus Koleolepas (Cirripedia:Thoracica: Koleolepadidae), with new records from Australian waters. Records of the Western Australian Museum 29:1–.Hurley, A. C. 1973. Fecundity of the Acorn Barnacle Balanus pacificus Pilsbry: A Fugitive Species. Limnology and Oceanography 18:386–93.Jeffries, W. B., H. K. Voris, and C. M. Yang. 1985. Growth of Octolasmis cor (Aurivillius, 1892) on the gills of Scylla serrata (Forskal, 2755). The Biological Bulletin 169:291–96.Jones, D., M. A. Hewitt, and A. Sampey. 2000. A checklist of the Cirripedia of the South China Sea. The Raffles bulletin of zoology 8:233–07.Jones, D., and A. M. Hosie. 2009. A new species of Calantica from Western Australian waters (Thoracica: Scalpellomorpha: Calanticidae). Records of the Western Australian Museum 25:239–46.Jones, D. S. 1991. A history of the discovery and description of Australian barnacles (Cirripedia: Thoracica), including a bibliography of reference works. Archives of Natural History 18:149–78.Jones, D. S. 1993. A New Neolepas (Cirripedia: Thoracica: Scalpellidae) from an Abyssal Hydrothermal Vent, Southeast Pacific. Bulletin of Marine Science 52:937–48.Jones, D. S., and A. M. Hosie. 2016. A checklist of the barnacles (Cirripedia: Thoracica) of Singapore and neighbouring waters. Raffles Bulletin of Zoology 34:241–11.Jones, L. W. G., and D. J. Crisp. 1954. The larval stages of the Barnacle Balanus improvisus Darwin. Proceedings of the Zoological Society of London 123:765–80.Kado, R., and R. Hirano. 1994. Larval development of two Japanese megabalanine barnacles, Megabalanus volcano (Pilsbry) and Megabalanus rosa (Pilsbry) (Cirripedia, Balanidae), reared in the laboratory. Journal of Experimental Marine Biology and Ecology 175:17–1.Karande, A. A. 1974. Larval development of the barnacle Tetraclitella karandei reared in the laboratory. The Biological Bulletin 146:249–57.Karande, A. A., and M. K. Thomas. 1971. Laboratory rearing of Balanus amphitrite communis (D.). Current Science 40:109–10.Kaufmann, R. 1965. Zur Embryonal-und Larvalentwicklung von Scalpellum scalpellum L.(crust. cirr.). Zoomorphology 55:161–32.Klepal, W. 1985. Ibla cumingi (Crustacea, Cirripedia) - A Gonochoristic Species (Anatomy, Dwarfing and Systematic Implications). Marine Ecology 6:47–19.Korn, O. M., and A. S. Elfimov. 1999. Larval development of a warm-water immigrant barnacle, Solidobalanus fallax (Cirripedia: Archaeobalanidae) reared in the laboratory. Journal of the Marine Biological Association of the United Kingdom 79:1039–044.Korn, O. M., A. S. Elfimov, and N. V. Skreptsova. 2001. Larval development of a barnacle, <span class="italic">Balanus spongicola</span> (Cirripedia: Balanidae) reared in the laboratory. Journal of the Marine Biological Association of the United Kingdom 81:775–79.Lang, W. H. 1979. Larval development of shallow water barnacles of  the Carolinas (Cirripedia: Thoracica) with keys to naupliar stage. NOAA Technical Report, NMFS Circular 421:1–9.Lang, W. H. 1976. The larval development and metamorphosis of the pedunculate barnacle Octolasmis mulleri (Coker, 1902) reared in the laboratory. Biol Bull 150:255–67.Lee, C., and C. H. Kim. 1991. Larval development of Balanus albicostatus Pilsbry (Cirripedia, Thoracica) reared in the laboratory. Journal of Experimental Marine Biology and Ecology 147:231–44.Lee, C., J. M. Shim, and C. H. Kim. 1999. Larval development of Balanus reticulatus Utinomi, 1967 (Cirripedia, Thoracica) and a comparison with other barnacle larvae. Journal of Plankton Research 21:2125–142.Lee, C., J. M. Shim, and C. H. Kim. 2000. Larval development of Capitulum mitella (Cirripedia: Pedunculata) reared in the laboratory. Journal of the Marine Biological Association of the United Kingdom 80:457–64.Leung, T. Y., and D. S. Jones. 2000. Barnacles (Cirripedia: Thoracica) from epibenthic substrata in the shallow offshore waters of Hong Kong. P. in B. Morton, ed. The Marine Flora and Fauna of Hong Kong and Southern China V. Hong Kong University Press, Hong Kong.Lewis, C. A. 1975. Some observations of factors affecting embryonic and larval growth of Pollicipes polymerus (Cirripedia: Lepadomorpha) in vitro. Mar. Biol. 32:127–39.Miller, K. M., S. M. Blower, D. Hedgecock, and J. Roughgarden. 1989. Comparison of Larval and Adult Stages of Chthamalus dalli and Chthamalus fissus (Cirripedia: Thoracica). Journal of Crustacean Biology 9:242–56.Miller, K. M., and J. Roughgarden. 1994. Descriptions of the larvae of Tetraclita rubescens and Megabalanus californicus with a comparison of the common barnacle larvae of the central California coast. Journal of Crustacean Biology 579–00.Molares, J., F. Tilves, and C. Pascual. 1994. Larval development of the pedunculate barnacle Pollicipes cornucopia (Cirripedia: Scalpellomorpha) reared in the laboratory. Marine Biology 120:261–64.Molenock, J., and E. D. Gomez. 1972. Larval Stages and Settlement of the Barnacle Balanus (Conopea) galeatus (L.) (Cirripedia Thoracica). Crustaceana 23:100–08.Moyse, J. 1963. A Comparison of the Value of Various Flagellates and Diatoms as Food for Barnacle Larvae. Journal du Conseil 28:175–87.Moyse, J. 1987. Larvae of lepadomorph barnacles. Pp. 329–62 in A. J. Southward, ed. Barnacle Biology. A. A. Balkema, Rotterdam.Namboothri, N., and S. A. Fernando. 2012. Intertidal Distribution of the Coral-Boring Barnacle Lithotrya nicobarica Reinhardt, 1850 in the Great Nicobar Island. Pp. 49–7 in K. Venkataraman, C. Raghunathran, and C. Sivaperuman, eds. Ecology of Faunal Communities on the Andaman and Nicobar Islands. Springer, Heidelberg, Germany.Newman, W. A. 1986. Origin of the Hawaiian marine fauna: Dispersal and vicariance. P. in R. H. Gore and K. L. Heck, eds. Crustacean biogeography. A. A. Belkema, Rotterdam/Boston.Newman, W. A., and A. Ross. 1971. Antarctic Cirripedia.Nogata, Y., and K. Matsumura. 2006. Larval development and settlement of a whale barnacle. Biology Letters 2:92–3.O’onnor, M. I., J. F. Bruno, S. D. Gaines, B. S. Halpern, S. E. Lester, B. P. Kinlan, and J. M. Weiss. 2007. Temperature control of larval dispersal and the implications for marine ecology, evolution, and conservation. Proceedings of the National Academy of Sciences 104:1266–271.Ozaki, Y., Y. Yusa, S. Yamato, and T. Imaoka. 2008. Reproductive ecology of the pedunculate barnacle Scalpellum stearnsii (Cirripedia: Lepadomorpha: Scalpellidae). Journal of the Marine Biological Association of the United Kingdom 88:77–3.Page, H. M. 1984. Local variation in reproductive patterns of two species of intertidal barnacles, Pollicipes polymerus Sowerby and Chthamalus fissus Darwin. Journal of experimental marine biology and ecology 74:259–72.Pannacciulli, F. G., G. Manetti, and F. Maltagliati. 2009. Genetic diversity in two barnacle species, Chthamalus stellatus and Tesseropora atlantica (Crustacea, Cirripedia), with different larval dispersal modes in the archipelago of the Azores. Marine Biology 156:2441–450.Pappalardo, P., F. B. Pitombo, P. A. Haye, and J. P. Wares. 2016. A rose by any other name: systematics and diversity in the Chilean Giant Barnacle Austromegabalanus Psittacus (Molina, 1782)(Cirripedia). Journal of Crustacean Biology 36:180–88.Peirano, A., C. Lombardi, N. Ponzè and S. Cocito. 2013. Verrucae on sea-fans: unexpected abundance of the barnacle Conopea calceola (Ellis) on the gorgonian Eunicella singularis (Esper) in the Mediterranean Sea. Rapp. Comm. int. Mer Méit. 40:586.Péez-Losada, M., M. Harp, J. T. Høg, Y. Achituv, D. Jones, H. Watanabe, and K. A. Crandall. 2008. The tempo and mode of barnacle evolution. Molecular Phylogenetics and Evolution 46:328–46.Pilsbry, H. A. 1907. The Barnacles (Cirripedia) Contained in the Collection of the U. S. National Museum.Pyefinch, K. A. 1948a. Methods of identification of the larvae of Balanus balanoides (L.), B. crenatus Brug. and Verruca stroemia OF Muller. Journal of the Marine Biological Association of the United Kingdom 27:451–63.Pyefinch, K. A. 1948b. Notes on the biology of cirripedes. Journal of the Marine Biological Association of the United Kingdom 27:464–03.Rainbow, P. S., J. Green, and P. Denny. 1989. A Note on the Intertidal Ecology of Two Little Known Barnacles, Balanus kondakovi Tarasov & Zevina, 1957, and Balanus patelliformis Bruguièe, 1789, from a Malaysian Mangrove Swamp (Cirripedia, Balanomorpha). Crustaceana 57:104–07.Rees, D. J., C. Noever, J. T. Hoeg, A. Ommundsen, and H. Glenner. 2014. On the origin of a novel parasitic-feeding mode within suspension-feeding barnacles. Current biology  CB 24:1429–4.Roux, P. J. L., G. M. Branch, and M. A. P. Joska. 1990. On the distribution, diet and possible impact of the invasive European shore crab Carcinus maenas (L.) along the South African coast. South African Journal of Marine Science 9:85–3.Sandison, E. 1954. The identification of the nauplii of some South African barnacles with notes on their life histories. Trans. roy. Soc. S. Afr. 34:69.Sewell, S. 1926. A study of Lithotrya nicobarica Reinhardt. Records of the Indian Museum 28:269–30.Silina, A. V., and I. I. Ovsyannikova. 2000. Variability in Morphology of the Shell of the Barnacle, Balanus rostratus, under Different Conditions of Growth (Cirripedia, Thoracica). Crustaceana 73:519–24.Southward, A. J. 1998. New observations on barnacles (Crustacea: Cirripedia) of the Azores region. Novas observaçõs sobre as cracas (Crustacea: Cirripedia) na regiã dos Açres 16A:11–7.Southward, A. j. 1958. The Zonation of Plants and Animals on Rocky Sea Shores. Biological Reviews 33:137–77.Southward, A., and D. Jones. 2003. A revision of stalked barnacles (Cirripedia: Thoracica: Scalpellomorpha: Eolepadidae: Neolepadinae) associated with hydrothermalism, including a description of a new genus and species from a volcanic seamount off Papua New Guinea. Marine Biodiversity 32:77–3.Stancyk, S. E. 1979. Reproductive ecology of marine invertebrates. Published for the Belle W. Baruch Institute for Marine Biology and Coastal Research by the University of South Carolina Press.Stewart, B. A., P. A. Cook, and Y. Achituv. 1989. Naupliar Stages of the Coral Inhabiting Barnacle Savignium Milleporum (Darwin) (Cirripedia: Pyrgomatidae) from the Gulf of Eilat, Red Sea. Bulletin of Marine Science 45:164–73.Strathmann, M. F. 1987. Reproduction and Development of Marine Invertebrates of the Northern Pacific Coast: Data and Methods for the Study of Eggs, Embryos, and Larvae. University of Washington Press.Thiyagarajan, V., T. Harder, and P.-Y. Qian. 2003. Combined effects of temperature and salinity on larval development and attachment of the subtidal barnacle Balanus trigonus Darwin. Journal of Experimental Marine Biology and Ecology 287:223–36.Thiyagarajan, V., V. P. Venugopalan, T. Subramoniam, and K. V. K. Nair. 1997. Description of the naupliar stages of Megabalanus tintinnabulum (Cirripedia: Balanidae). Journal of Crustacean Biology 17:332–42.Tunnicliffe, V., and A. J. Southward. 2004. Growth and breeding of a primitive stalked barnacle Leucolepas longa (Cirripedia: Scalpellomorpha: Eolepadidae: Neolepadinae) inhabiting a volcanic seamount off Papua New Guinea.Utinomi, H. 1967. Occurence of a new pedunculate cirriped on a small spanish lobster Scyllarus bicuspidatus (De Man) from Kamae Bay, Northeastern Kyusyu. Publications of the Seto Marine Biological Laboratory 15:117–20.Venegas, R. M., V. Ortiz, A. Olguin, and S. A. Navarrete. 2000. Larval Development of the Intertidal Barnacles Jehlius cirratus and Notochthamalus scabrosus (Cirripedia: Chthamalidae) under Laboratory Conditions on JSTOR.Voris, H. K., and W. B. Jeffries. 2001. Distribution and size of a stalked barnacle (Octolasmis muelleri) on the blue crab, Callinectes sapidus. Bulletin of Marine Science 68:181–90.Wares, J. P., M. S. Pankey, F. Pitombo, L. G. Daglio, and Y. Achituv. 2009. A “hallow Phylogeny”of Shallow Barnacles (Chthamalus). PLoS ONE 4:e5567.Watanabe, H., J. T. Høg, B. K. K. Chan, R. Kado, S. Kojima, and A. Sari. 2008. First report of antennular attachment organs in a barnacle nauplius larva. Journal of Zoology 274:284–91.Watanabe, H., R. Kado, M. Kaida, S. Tsuchida, and S. Kojima. 2006. Dispersal of vent-barnacle (genus Neoverruca) in the Western Pacific. Cahiers de Biologie Marine 47:353–57.Watanabe, H., R. Kado, S. Tsuchida, H. Miyake, M. Kyo, and S. Kojima. 2004. Larval development and intermoult period of the hydrothermal vent barnacle <span class="italic">Neoverruca</span> sp. Journal of the Marine Biological Association of the United Kingdom 84:743–45.Watanabe, H., S. Tsuchida, K. Fujikura, H. Yamamoto, F. Inagaki, M. Kyo, and S. Kojima. 2005. Population history associated with hydrothermal vent activity inferred from genetic structure of neoverrucid barnacles around Japan. Marine Ecology Progress Series 288:233–40.Wijayanti, H., Y. Yusa, and R. Kado. 2017. Larval development of the epizoic barnacle Octolasmis unguisiformis Kobayashi & Kato, 2003 (Cirripedia, Pedunculata). Crustaceana 90:321–36.Wirtz, P., R. Araúo, and A. J. Southward. 2006. Cirripedia of Madeira. Helgoland Marine Research 60:207–12.Yamaguchi, T. 1973. On Megabalanus (Cirripedia, Thoracica) of Japan. Publications of the Seto Marine Biological Laboratory 21:115–40.Yamaguchi, T., W. A. Newman, and J. Hashimoto. 2004. A cold seep barnacle (Cirripedia: Neolepadinae) from Japan and the age of the vent/seep fauna. Journal of the Marine Biological Association of the United Kingdom 84:111–20.Yan, Y. 2003. Larval development of the barnacle Chinochthamalus scutelliformis (Cirripedia: Chthamalidae) reared in the laboratory. Journal of Crustacean Biology 23:513–21.Yan, Y., and B. K. Chan. 2001. Larval development of Chthamalus malayensis (Cirripedia: Thoracica) reared in the laboratory. Journal of the Marine Biological Association of the United Kingdom 81:623–32.Yan, Y., C. Haoru, H. Liangmin, and S. Lihua. 2005. Larval development of the barnacle Ibla cumingi (Cirripedia: Pendunculata: Iblidae) reared in the laboratory. Journal of The Marine Biological Association of The United Kingdom - J MAR BIOL ASSN UK 85:903–07.Yang, Y. 2003. Larval Development of the Barnacle Chinochthamalus scutelliformis (Cirripedia: Chthamalidae) Reared in the Laboratory. Journal of Crustacean Biology 23:513–21.Yorisue, T., R. Kado, H. Watanabe, J. T. Høg, K. Inoue, S. Kojima, and B. K. K. Chan. 2013. Influence of water temperature on the larval development of Neoverruca sp. and Ashinkailepas seepiophila—mplications for larval dispersal and settlement in the vent and seep environments. Deep Sea Research Part I: Oceanographic Research Papers 71:33–7.Young, J. S. 1990. Lepadomorph cirripeds from the Brazilian coast. I. - Families Lepadidae, Poecilasmatidae and Heteralepadidae. Bulletin of Marine Science 47:641–55.Young, P. S. 2001. Deep-sea Cirripedia Thoracica (Crustacea) from the northeastern Atlantic collected by French expeditions. ZOOSYSTEMA-PARIS- 23:705–56.Young, P. S. 2002. Revision of the Scapellidae (Crustacea, Cirripedia) in the collection of the Museum national d’istoire naturelle, France, studied by Abel Gruvel. ZOOSYSTEMA-PARIS- 24:309–46.Yusa, Y., S. Yamato, M. Kawamura, and S. Kubota. 2015. Dwarf males in the barnacle Alepas pacifica Pilsbry, 1907 (Thoracica, Lepadidae), a symbiont of jellyfish. Crustaceana 88:273–82.Yusa, Y., S. Yamato, and M. Marumura. 2001. Ecology of a parasitic barnacle, Koleolepas avis: relationship to the hosts, distribution, left–ight asymmetry and reproduction. Journal of the Marine Biological Association of the UK 81:781–88.Zabin, C., J. Zardus, F. Pitombo, V. Fread, and M. Hadfield. 2007. A tale of three seas: consistency of natural history traits in a Caribbean–tlantic barnacle introduced to Hawaii. Biol Invasions 9:523–44.Zann, L. P., and B. M. Harker. 1978. Egg Production of the Barnacles Platylepas ophiophilus Lanchester, Platylepas hexastylos (O. Fabricius), Octolasmis warwickii Gray and Lepas anatifera Linnaeus. Crustaceana 35:206–14.Zardus, J. D., and M. G. Hadfield. 2004. Larval Development and Complemental Males in Chelonibia testudinaria, a Barnacle Commensal with Sea Turtles. Journal of Crustacean Biology 24:409–21.Zevina, G. B. 1972. Benthic Lepadomorpha (Cirripedia Thoracica) from the Southeast Pacific. Crustaceana 22:39–3.Zevina, G. B. 1974. The Cirripedia Thoracica of the Kerguelen Islands. Crustaceana 27:209–15.Zintzen, V., and F. Kerckhof. 2009. The sponge-inhabiting barnacle Acasta spongites (Poli, 1795) (Crustacea, Cirripedia), a first record for the southern North Sea: how artificial habitats may increase the range of a species. Belg. J. Zool 139:166–68.
